# Global scale transcriptome analysis of *Arabidopsis* embryogenesis *in vitro*

**DOI:** 10.1186/s12864-015-1504-6

**Published:** 2015-04-16

**Authors:** Anushka M Wickramasuriya, Jim M Dunwell

**Affiliations:** School of Agriculture, Policy and Development, University of Reading, Reading, UK

**Keywords:** Somatic embryogenesis, Transcriptomics, RNA-Seq, *Arabidopsis thaliana*, DNA replication, Cell cycle, Epigenetics

## Abstract

**Background:**

Somatic embryogenesis (SE) in plants is a process by which embryos are generated directly from somatic cells, rather than from the fused products of male and female gametes. Despite the detailed expression analysis of several somatic-to-embryonic marker genes, a comprehensive understanding of SE at a molecular level is still lacking. The present study was designed to generate high resolution transcriptome datasets for early SE providing the way for future research to understand the underlying molecular mechanisms that regulate this process. We sequenced *Arabidopsis thaliana* somatic embryos collected from three distinct developmental time-points (5, 10 and 15 d after *in vitro* culture) using the Illumina HiSeq 2000 platform.

**Results:**

This study yielded a total of 426,001,826 sequence reads mapped to 26,520 genes in the *A. thaliana* reference genome. Analysis of embryonic cultures after 5 and 10 d showed differential expression of 1,195 genes; these included 778 genes that were more highly expressed after 5 d as compared to 10 d. Moreover, 1,718 genes were differentially expressed in embryonic cultures between 10 and 15 d. Our data also showed at least eight different expression patterns during early SE; the majority of genes are transcriptionally more active in embryos after 5 d. Comparison of transcriptomes derived from somatic embryos and leaf tissues revealed that at least 4,951 genes are transcriptionally more active in embryos than in the leaf; increased expression of genes involved in DNA cytosine methylation and histone deacetylation were noted in embryogenic tissues. *In silico* expression analysis based on microarray data found that approximately 5% of these genes are transcriptionally more active in somatic embryos than in actively dividing callus and non-dividing leaf tissues. Moreover, this identified 49 genes expressed at a higher level in somatic embryos than in other tissues. This included several genes with unknown function, as well as others related to oxidative and osmotic stress, and auxin signalling.

**Conclusions:**

The transcriptome information provided here will form the foundation for future research on genetic and epigenetic control of plant embryogenesis at a molecular level. In follow-up studies, these data could be used to construct a regulatory network for SE; the genes more highly expressed in somatic embryos than in vegetative tissues can be considered as potential candidates to validate these networks.

**Electronic supplementary material:**

The online version of this article (doi:10.1186/s12864-015-1504-6) contains supplementary material, which is available to authorized users.

## Background

Somatic embryogenesis (SE), where a single or a group of somatic cells differentiate to form embryonic cells under suitable *in vitro* conditions [1] is a good system to explore gene expression patterns associated with initial stages of embryo development. The formation of embryos from somatic cells closely resembles the developmental pathway of zygotic embryos (ZEs) and hence, the molecular information generated for the SE pathway could be used to explain the dynamic molecular interactions that take place during early embryogenesis [1,2]. Recently, a broad analysis of SE regulation in higher plants with an especial emphasis on associated developmental pathways, differential gene expression, and proteomics has been reviewed elsewhere [3,4].

Over the past few years, progress of molecular techniques have immensely contributed in understanding the molecular aspects of SE in many plant species i.e. *Arabidopsis* [5,6], cotton [7], alfalfa [8], conifer [9], potato [10], *Glycine max* [11], oil palm [12,13], maize [14], *Picea glauca* [15], *P. balfouriana* [16], *Vitis vinifera* [17], *Medicago truncatula* [18] and *Manihot esculenta* [19,20]. Although the draft molecular interaction network provided for SE highlights the potential interactions at a molecular level [21], the functional characterization of the majority of genes involved in somatic-to-embryogenic transition and subsequent embryo maturation still remain largely unknown.

The advent of high throughput genomic and transcriptomic approaches has created great interest in the development of networks for plant metabolic processes to study how genes or gene products are regulated spatially and temporally to achieve cellular demands. Studies based on large scale transcriptome profiling have given a fundamental insight into the aspects of co-expressing genes and their roles in metabolic pathways. For instance, gene expression during the course of *Arabidopsis* ZE development, from zygote to mature embryos have been studied using microarray technology by Xiang et al. [22]. Recently, a global scale transcriptomic profiling of developing embryos using RNA sequencing (RNA-Seq) has been reported for the monocot model plant, *Oryza sativa* [23]. Moreover, Illumina RNA-Seq platform has been successfully used in a study related to developing seeds of maize [24].

Although, large scale genetic resources have been generated for *Arabidopsis* ZE development, a high resolution dataset for SE is still not available. Such a dataset is required to develop a system level model which will facilitate better understating of the molecular aspects of embryo development *in vitro*. Therefore, the present study was designed to generate transcript datasets for early stage somatic embryos through high-throughput Illumina HiSeq 2000. These data may provide a solid framework for future studies to investigate molecular interactions in early SE.

## Results and discussion

Understanding the molecular mechanisms that drives plant embryogenesis is a major challenge due to limited accessibility to the developing embryos i.e. early stages of *Arabidopsis* ZEs. Given the advantage of high-throughput sequencing systems as well as the advanced knowledge of the ZE development pathway, availability of a standard protocol to induce somatic embryos and accessibility to the well annotated genome information, directed us to select the model plant, *Arabidopsis*, to study the global gene expression patterns in plant SE through RNA-Seq. The transcriptome information given here would serve as a useful resource in future to develop a regulatory system for this dicot model species; this will pave the way for genetic improvement of SE in other dicot species.

### Illumina sequencing and mapping sequence reads to the reference genome

Transcriptome sequencing through RNA-Seq is a relatively straight forward approach that allows exploring gene expression profiles at a global scale. To provide a comprehensive overview of *Arabidopsis* SE at a transcriptional level, we sequenced cDNA libraries constructed from three distinct *in vitro* embryo developmental time-points using the Illumina HiSeq 2000 platform. This produced a total of 605,027,558 sequence reads, encompassing 30,251 Mbases from all four cDNA libraries, embryos after 5 d (SE_5D), 10 d (SE_10D), 15 d (SE_15D) of *in vitro* culture and wild type (WT) leaf tissues (WT_L). On average 90.8% of the quality filter passed reads generated for all three somatic embryo samples were mapped uniquely to the reference genome. Another 2.4% of reads were mapped to multiple locations in the genome and the remaining reads were either unmapped (2.1%) or did not show primary hits (4.7%). Of the mapped reads identified in both tissue types, on average approximately 98.5% of reads were mapped to the exons, 0.9% of reads were mapped to introns and the remaining 0.5% of reads were aligned with the 10 kb upstream and downstream of the transcripts. A summary of mapping statistics obtained for each sample is given in Table 1.

**Table 1:** **Mapping statistics for quality filtered reads generated for embryogenic and leaf tissues**

	**SE_5D**	**SE_10D**	**SE_15D**	**WT_L**
**Total number of QC-passed reads**	73882504	212208700	139910622	179025732
**Total Number of mapped reads**	75839181	222139250	148769022	183167937
**Mapped percentage (%)**	97.82	98.18	97.24	99.10
Read −1*	37996562	111267307	74522675	91775211
Read −2*	37842619	110871943	74246347	91392726
**Total number of unmapped reads**	1613269	3862501	2560237	1613269
**Un-mapped percentage (%)**	2.18	1.82	2.76	0.90

*read-1 and read-2: forward and reverse primer derived reads.

**Figure 1:**
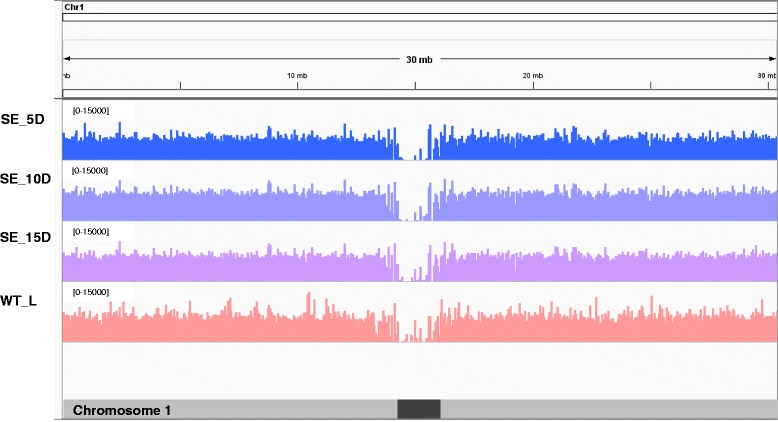
Coverage plots generated for transcripts aligned to the chromosome 1. SE_5D, SE_10D and SE_15D are to represent embryogenic samples collected after 5, 10 and 15 d of *in vitro* culture, respectively. WT_L: WT leaf tissues.

The average coverage profiles computed for the mapped reads using the Integrative Genomic Viewer (IGV) tool for each chromosome showed that the read coverage of each chromosome is uniform across all samples examined. Log transformed (base 2) coverage values were used to generate coverage plots as it gives a better visualization of read depths. Only the chromosome 1 coverage map is shown here (Figure 1).

**Figure 2:**
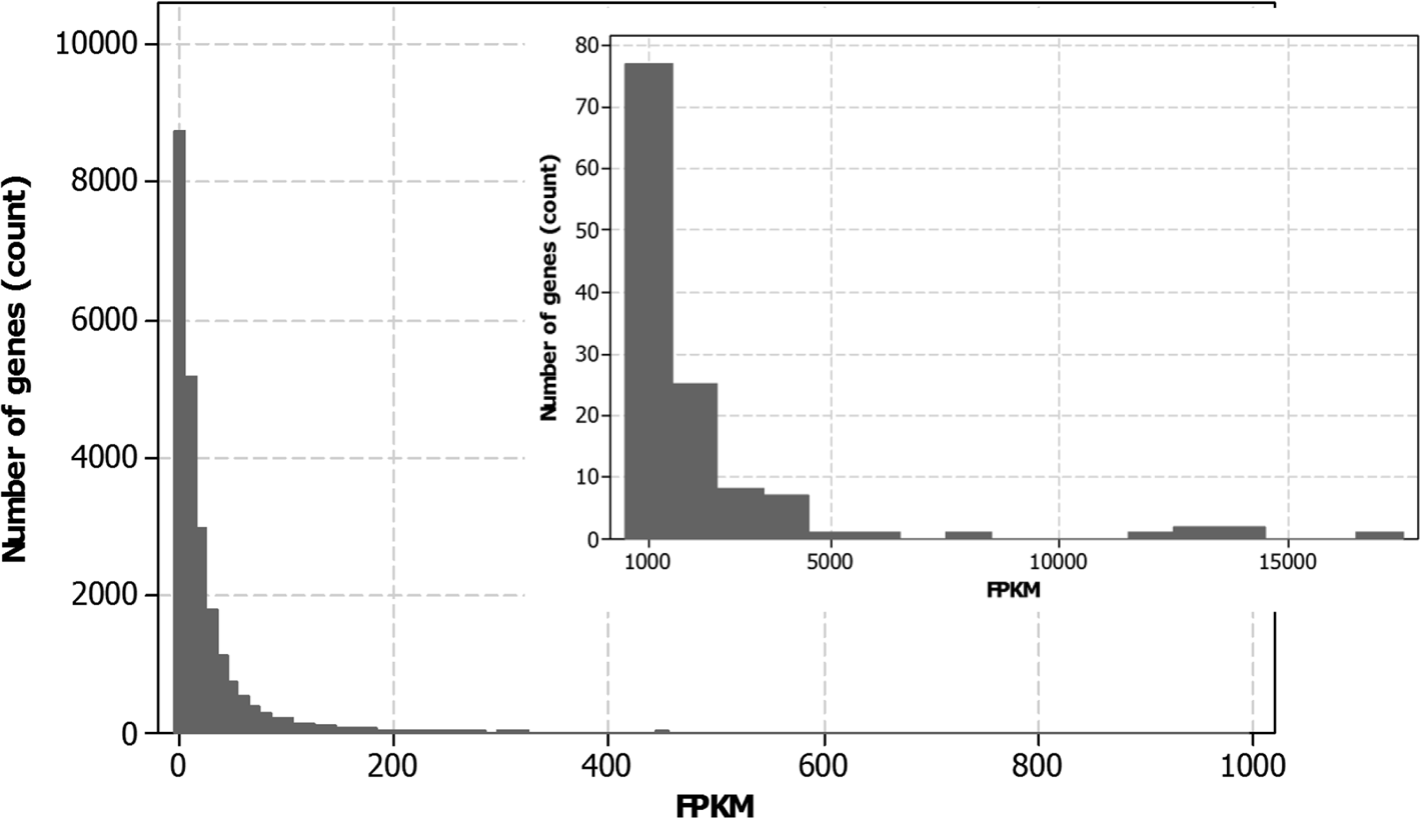
Distribution of transcript abundance in somatic embryo transcriptomes. This represents the transcriptome of embryogenic tissues collected after 5 d of *in vitro* culture.

Conversion of mapped and assembled read counts into normalized digital transcript levels (Fragments Per Kilobase of exon per Million fragments mapped (FPKM)) is a prerequisite for comparing expression profiles of genes within or between samples to provide a comprehensive overview of transcriptomes. For downstream gene expression analysis, expression measures of individual gene isoforms were combined to obtain the final transcript level for a particular gene. In this study we report the presence of transcripts for 26,520 annotated genes in at least one of the three somatic embryo samples examined; this reflects approximately ≈ 80% of the annotated genes reported in the latest *Arabidopsis* genome release (Additional file 1). Among these genes, 24,097 of protein coding genes (91%), 1,338 (5%) transposable elements (TEs), 466 (2%) pseudo genes and 619 (2%) other RNA genes were identified. The distribution of FPKM values for each sample analysed showed a similar pattern, skewing to the right (Figure 2).

**Figure 3:**
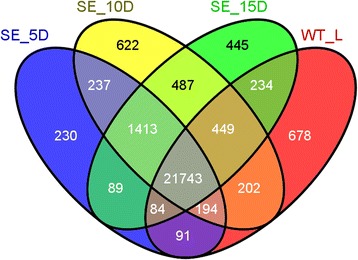
Venn diagram representing the transcribed genes detected in embryogenic and leaf tissues. SE_5D, SE_10D and SE_15D are to represent embryogenic samples collected after 5, 10 and 15 d of *in vitro* culture, respectively. WT_L: WT leaf tissues.

In brief, 24,081 (73%), 25,347 (77%), 24,944 (75%) and 23,675 (72%) annotated genes were transcriptionally active in SE_5D, SE_10D, SE_15D and WT_L, respectively. The Figure 3 summarizes the distribution of expressed genes found in each sample examined. Based on our study, it was noted that a total of 3,523 genes (2,469 protein coding genes and 723 TEs) are expressed only in embryogenic tissues but not in WT leaf tissues. This included 200 transcription factor (TF) encoding genes over 31 TF families. MYB (30 genes), MADS-box (23), C3H (19), basic helix-loop-helix (bHLH) (18), C2H2 (17), homeobox (12) and APETALA2 (AP2)/ethylene responsive element binding proteins (EREBP) (11) were the main TF families observed.

**Figure 4:**
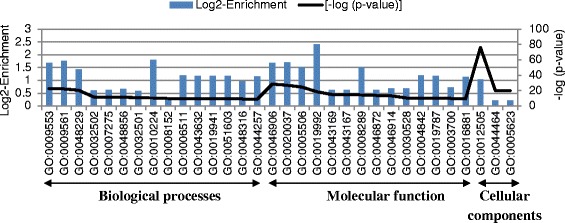
More highly enriched GO terms found within the gene subset that showed a somatic embryo specific expression pattern. GO:0009553: embryo sac development; GO:0009561:megagametogenesis; GO:0048229: gametophyte development; GO:0032502: developmental process; GO:0007275: multicellular organismal development; GO:0048856: anatomical structure development; GO:0032501: multicellular organismal process; GO:0010224: response to UV-B; GO:0008152: metabolic process; GO:0006511: ubiquitin-dependent protein catabolic process; GO:0043632: modification-dependent macromolecule catabolic process; GO:0019941: modification-dependent protein catabolic process; GO:0051603: proteolysis involved in cellular protein catabolic process; GO:0048316: seed development; GO:0044257: cellular protein catabolic process; GO:0046906: tetrapyrrole binding; GO:0020037: heme binding; GO:0005506: iron ion binding; GO:0019992: diacylglycerol binding; GO:0043169: cation binding; GO:0043167: ion binding; GO:0008289: lipid binding; GO:0046872: metal ion binding; GO:0046914: transition metal ion binding; GO:0030528: transcription regulator activity; GO:0004842: ubiquitin-protein ligase activity; GO:0019787: small conjugating protein ligase activity; GO:0003700: transcription factor activity; GO:0016881: acid-amino acid ligase activity; GO:0012505: endomembrane system; GO:0044464: cell part; GO:0005623: cell.

Furthermore, the Gene Ontology (GO) enrichment analysis of this gene subset identified 1,864 genes with annotated GO terms. They were significantly enriched for 132 GO terms over three main functional categories at p < 0.05, biological processes (BP) (93), molecular function (MF) (31) and cellular components (CC) (8). The highly enriched GO terms (p < 10^−9^) found in each main functional category is shown in Figure 4. For instance, embryo sac development (GO:0009553), megagametogenesis (GO:0009561), gametophyte development (GO:0048229), developmental process (GO:0032502), tetrapyrrole binding (GO:0046906), heme binding (GO:0020037), iron ion binding (GO:0005506), endomembrane system (GO:0012505), cell part (GO:0044464) and cell (GO:0005623) were the main functional sub-groups that showed highest significance. To determine the biological pathways that are functionally more active, the specifically transcribed gene subset (3,523) found in somatic embryos was further analysed through the SkyPainter tool (*Arabidopsis* Reactome). This showed over-representation of 17 main biological pathways (Table 2).

**Table 2:** **Over-represented biological pathways found within the specifically transcribed gene subset detected in embryogenic tissues**

**Plant biological pathway**	**Probability**
Glucosinolate biosynthesis from tryptophan	2.50E-03
Glucosinolate biosynthesis from phenylalanine	2.70E-03
Glucosinolate biosynthesis from homomethionine	2.70E-03
Anthocyanin biosynthesis	2.90E-03
Gibberellin biosynthesis III (early C-13 hydroxylation)	3.30E-03
Cytokinins-O-glucoside biosynthesis	4.10E-03
Cytokinins 7-N-glucoside biosynthesis	4.10E-03
Cytokinins 9-N-glucoside biosynthesis	4.10E-03
ent-kaurene biosynthesis	4.30E-03
(deoxy)ribose phosphate degradation	1.40E-02
Abscisic acid biosynthesis	1.80E-02
de novo biosynthesis of purine nucleotides II	1.90E-02
Gibberellin biosynthesis I (non C-3, non C-13 hydroxylation)	2.10E-02
Gibberellin biosynthesis II (early C-3 hydroxylation)	2.10E-02
Gibberellin inactivation	2.10E-02
de novo biosynthesis of purine nucleotides I	7.60E-02
Trans-zeatin biosynthesis	9.50E-02

In general, as compared to previous plant embryogenesis related studies, our study report abundant transcript information for downstream analysis. For example, an expression analysis based on microarray technique has demonstrated expression of at least 22,800 genes across the three embryonic stages, globular, heart and torpedo in *Arabidopsis* [25]. A similar expression analysis has shown expression of 17,594 different transcripts in at least one of the stages or sub-regions of *Arabidopsis* developing seeds [26]. Therefore, the *Arabidopsis* somatic embryo transcriptome information given here may serve as a valuable resource for future studies.

### Gene expression patterns across the three distinct *in vitro* embryo developmental time-points

To provide an overview of potential gene expression patterns that may exist during *in vitro* embryogenesis, transcript levels of 26,520 annotated genes were compared between different embryo developmental time-points. Of these, 23,156 genes were co-expressed in all three developmental time-points; 431 were co-expressed between SE_5D and SE_10D; 936 were co-expressed between SE_10D and SE_15D; 173 were co-expressed between SE_5D and SE_15D. In addition, 321, 824 and 679 genes were preferentially expressed in SE_5D, SE_10D and SE_15D, respectively. Although a considerable fraction of co-expressed genes was identified between time-points, the most of the genes showed noticeable variations in transcript levels. Thus, to determine differentially expressed genes (DEGs), transcript levels (FPKM) of genes were compared between the time-points. A total of 1,195 DEGs were found between 5 and 10 d after *in vitro* culture with 417 up-regulated (Log2 [fold change (FC)] ≥ 2.0) and 778 down-regulated (Log2 [FC] ≤ −2.0) genes. It was found that 82 (7%) TF encoding genes are differentially expressed between these two time-points and the majority were from TF families such as MYB (14), bHLH (12) and MADS (9) (Table 3). Additionally, several embryogenesis related genes such as *LATE EMBRYOGENESIS ABUNDANT* (*LEA*) genes (*AT1G54890*, *AT1G64065*, *AT3G19430*, *AT2G35970*, *AT4G27400*, *AT1G61760*, *AT2G40170*, *AT5G60530* and *AT5G54370*), nitrate transport 1.6 (A*T1G27080*), *AGAMOUS-like 81* (*AGL81*; *AT5G39750*), *MATERNAL EFFECT EMBRYO ARREST 27* (*AT2G34880*), *MATERNAL EFFECT EMBRYO ARREST 8* (*AT1G25310*), *ATS3-*like gene (*AT5G62210*), *EMBRYO SAC DEVELOPMENT ARREST 36* (*AT4G13890*), *ATS1* (*AT4G26740*) and *EMBRYO SAC DEVELOPMENT ARREST 24* (*AT1G70540*) were also detected among the DEGs.

**Table 3:** **Top most differentially expressed TF encoding genes detected between embryo developmental time-points**

**Gene ID**	**Log2 [FC]**	**TF family**	**Gene description**
***Differentially expressed TFs between SE_5D and SE_10D***			
*AT5G27090*	4.82	MADS	Protein agamous-like 54
*AT3G46070*	3.58	C2H2	C2H2-type zinc finger family protein
*AT5G52600*	3.52	MYB	MYB82
*AT5G40220*	3.28	MADS	Protein agamous-like 43
*AT1G66420*	3.27	GeBP	DNA-binding storekeeper protein-related transcriptional regulator
*AT1G13260*	3.26	RAV	AP2/ERF and B3 domain-containing TF
*AT2G47190*	3.09	MYB	R2R3 MYB DNA binding domain TF (MYB2)
*AT3G56970*	3.01	bHLH	ORG2 (BHLH038)
*AT5G41570*	3.00	WRKY	WRKY transcription factor 24
*AT5G15150*	2.83	Homeobox	Homeobox-leucine zipper protein HAT7 (HB-3)
*AT3G11110*	−5.91	C3H	RING-H2 finger protein ATL66
*AT4G09960*	−4.50	MADS	Agamous-like MADS-box protein AGL11
*AT1G75250*	−4.06	MYB-related	Protein RADIALIS-like 6
*AT1G25310*	−3.79	bHLH	Transcription factor MEE8
*AT2G27940*	−3.73	C3H	RING-H2 finger protein ATL57
*AT1G19510*	−3.65	MYB-related	Protein RADIALIS-like 5
*AT1G56650*	−3.64	MYB	Transcription factor MYB75
*AT3G06120*	−3.23	bHLH	Transcription factor MUTE
*AT5G46830*	−3.22	bHLH	Calcium-binding transcription factor NIG1
*AT5G53200*	−3.21	MYB-related	Transcription factor TRY
***Differentially expressed TFs between SE_10D and SE_15D***			
*AT2G21900*	6.41	WRKY	WRKY DNA-binding protein 59
*AT5G01900*	5.04	WRKY	WRKY DNA-binding protein 62
*AT2G40750*	4.80	WRKY	WRKY DNA-binding protein 54
*AT5G65790*	4.78	MYB	MYB domain protein 68
*AT1G74080*	4.28	MYB	MYB domain protein 122
*AT1G02230*	4.28	NAC	NAC domain containing protein 4
*AT2G45660*	4.27	MADS	AGAMOUS-like 20; Involved in controlling flowering
*AT5G22570*	4.17	WRKY	WRKY DNA-binding protein 38
*AT5G64810*	4.16	WRKY	WRKY DNA-binding protein 51
*AT1G66600*	4.13	WRKY	WRKY transcription factor 63; Involved in regulation of plant responses to ABA and drought stress
*AT2G34820*	−15.05	bHLH	Basic helix-loop-helix 53
*AT5G39750*	−7.70	MADS	AGAMOUS-like 81
*AT1G24260*	−4.41	MADS	MADs box transcription factor SEPALLATA3
*AT3G50330*	−3.80	bHLH	HECATE 2
*AT4G00120*	−3.67	bHLH	INDEHISCENT
*AT5G14010*	−3.21	C2H2	Zinc finger protein KNUCKLES; Mediates the repression of WUS in floral meristem determinacy control
*AT4G30180*	−3.00	bHLH	Hypothetical protein
*AT4G26150*	−3.00	C2C2-Gata	Putative GATA transcription factor 22
*AT1G25250*	−2.83	C2H2	Indeterminate-domain 16
*AT4G29030*	−2.82	Trihelix	Putative membrane lipoprotein

A similar differential gene expression analysis between 10 and 15 d after *in vitro* cultures identified a total of 1,067 up regulated genes and 651 down regulated genes. This comprised 114 (7%) TF genes; the majority were members of TF families such as MYB or MYB related (20 genes), MADS-box (12) and AP2/EREBP (12) (Table 3). Furthermore, *LEA* genes such as *AT1G61760*, *AT5G22870*, *AT5G53730, AT3G02480*, *AT1G52680*, *AT5G54370*, *AT4G36600*, *AT3G22500*, *AT5G06760*, *AT3G15670*, *AT2G35300*, *AT4G27400*, *AT1G32560* and *AT1G72100* as well as several other embryogenesis related genes i.e. *EMBRYO SAC DEVELOPMENT 39*, *SEED GENE 3* (*ATS3*), *AGL81*, *RESPONSIVE TO ABSCISIC ACID 28* (*RAB28*; *AT1G03120*) were also differentially expressed between SE_10D and SE_15D.

**Figure 5:**
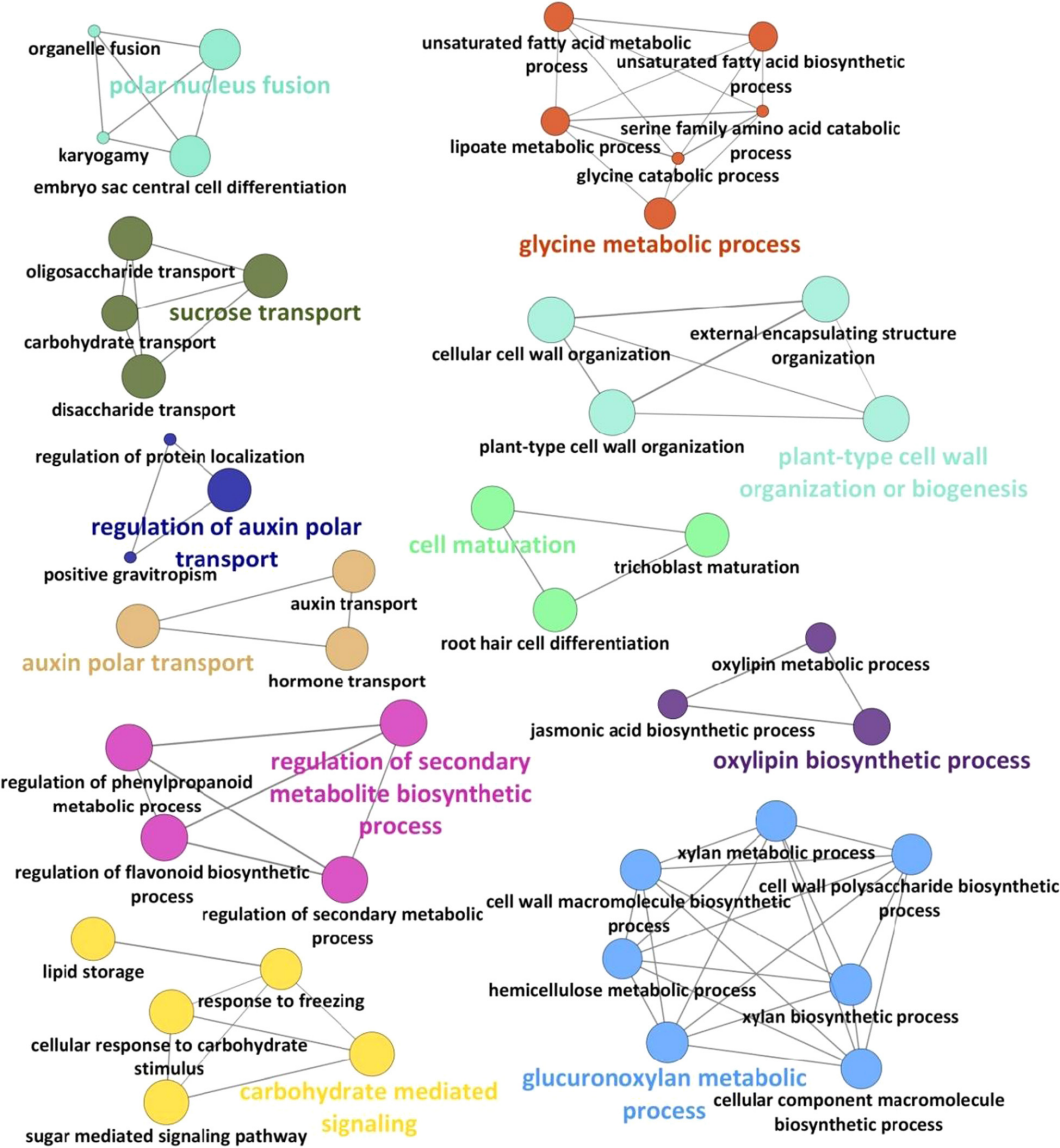
Illustration of functional networks based on enriched GO terms for BP for more highly expressed genes detected in embryogenic tissues after 5 d of *in vitro* culture as compared to 10 d. Networks were built using high stringency (kappa score = 0.8, GO level: 3 – 8). Only highly significant networks are presented here. The leading group term is based on the highest significance. Each node represents an enriched GO term whereas edges denote the functional relationship between the BP. Functionally related nodes are presented in the same colour.

Visualization of functional networks generated based on enriched GO terms related to BP for the identified DEGs between different somatic embryo developmental time-points exhibited the complexity of responses between the time-points. For example, genes expressed at a noticeably higher level in embryogenic tissues after 5 d as compared 10 d were mainly enriched for functional networks related to hormone transport (i.e. regulation of polar auxin transport), carbohydrate mediated signalling pathways and cell wall organization and biogenesis (Figure 5). In contrast, more highly expressed genes found in embryogenic cultures after 10 d were mainly enriched for functional networks related to energy metabolism and regulation of various responses to stimuli. A similar analysis of genes up regulated between embryogenic tissues after 10 and 15 d showed over-representation of biological networks such as cellular hormone biosynthesis and metabolic process (i.e. auxin, abscisic acid, and ethylene), regulation of secondary metabolic process and regulation of reproductive process.

**Figure 6:**
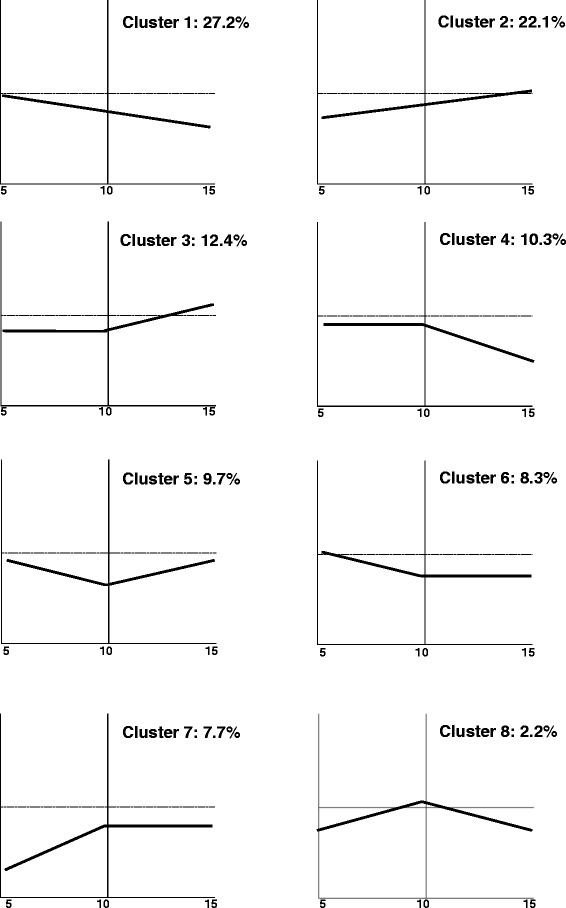
Potential gene expression patterns during SE. The X-axis represents somatic embryo developmental time-points, 5, 10 and 15 d after *in vitro* culture.

In summary, a total of 3,790 DEGs were detected in the process of *in vitro* embryo development; 1,195 genes between SE_5D and SE_10D; 1,718 genes between SE_10D and SE_15D; 2,817 genes between SE_5D and SE_15D. The remaining genes showed relatively stable expression during this process. In order to determine the potential gene expression patterns that may exist throughout the course of embryo development, the FPKM read counts of these DEGs were obtained for each embryo developmental time-point and transformed into a log (base 2) scale. Of these, 438 genes were excluded from the downstream cluster analysis as they were not expressed/detected (FPKM = 0) in at least one of the tissue sampling time-points. The remaining 3,352 genes were grouped into 10 user defined clusters based on their expression patterns. Based on the 10 clusters generated from the software, at least eight distinct gene expression patterns were detected (Figure 6). In accordance with previous research on rice embryo development [23], the majority of genes (912) fell into the continuously down regulated gene cluster (cluster 1). These genes were highly enriched for GO terms related to carbohydrate, lipid and amino acid metabolic processes. In addition, we found that expression levels of 740 genes gradually increase with embryo maturation (cluster 2). This gene subset was mainly enriched for stress responsive genes.

**Figure 7:**
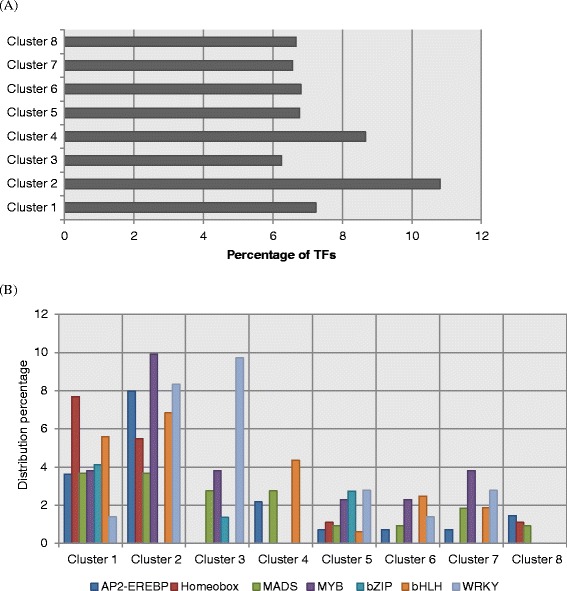
Distribution of TFs across the eight gene clusters. **(A)**, Percentage of TFs in each gene cluster; **(B)**, Distribution of TF family members across the clusters.

The eight different expression patterns identified here were further analyzed to examine whether any of these clusters are enriched for a particular TF family. It was found that at least 265 TF genes covering ≈ 30 different TF families are differentially expressed during SE. Figure 7A shows the distribution of TF related genes among the clusters. With the exception of clusters 2 and 4, almost all the clusters consisted of approximately 6-7% of TF related genes. A relatively higher percentage of TF related genes was detected in cluster 2 and 4, which was about 11% and 9%, respectively. Moreover, among the differentially expressed TFs detected, the majority belong to the TF families such as bHLH (35; 13%), MYB (34; 13%), AP2/EREBP (24; 9%), MADS-box (19; 7%), WRKY (19: 7%) and homeobox domain proteins (14; 5%). The distribution of highly occurring TF families within each cluster is highlighted in Figure 7B. The distribution percentage of each TF family was calculated in relation to each TF family size given in the *Arabidopsis* Gene Regulatory Information Server (AGRIS, http://arabidopsis.med.ohio-state.edu/). Most of the genes related to AP2/EPEBP, homeobox, MADS-box and bHLH families were found in clusters 1 and 2 where the gene expression was relatively high at the early and late stages, respectively.

Analysis of the remaining 438 genes that showed zero FPKM in at least one of the three embryo developmental time-points mainly fell into three groups: differential gene expression only between SE_5D and SE_10 (cluster A); differential gene expression only between SE_10D and SE_15D (cluster B); differential gene expression between SE_5D and SE_15D (cluster C). In summary, a total of 131 (cluster A), 244 (cluster B) and 63 (cluster C) genes were manually grouped into each cluster. It was found that genes related to anatomical structure development processes and catalytic activity were mainly enriched within the cluster A genes whereas genes related to peptide biosynthetic processes and nucleic acid binding were predominately enriched within cluster B. The genes fell into cluster C were largely enriched for stress responses. Additionally, these gene clusters were further analysed to examine whether any of these clusters are enriched for a particular family of TFs. Although several genes related to MADS-box, WRKY, MYB-related and homeobox was found within the clusters, none of the clusters showed enrichment for a particular type of TF family. This may be due to the presence of limited number of member genes within each cluster.

### Genes identified as being more highly expressed in embryogenic tissues than in leaf tissues

Although, a considerable fraction of co-expressed genes was detected between somatic embryos and WT leaf tissues, the majority of those genes showed a greater variation in transcript levels. Therefore, to identify more highly expressed genes in somatic embryos, transcriptomes of *in vitro* embryos and WT_L were compared and genes with Log2 ratios ≥ 2.0 were considered as more highly expressed genes in embryos. Based on this criterion, a total of 4,951 highly expressed genes were identified in somatic embryos; including 4,738 protein coding genes and 72 TEs. Of these, 3,896 genes were identified with annotated GO terms, enriching for 288 functional groups at p < 0.05. GO terms such as cellular process (GO: 0009987), structural constituent of ribosome (GO: 0003735) and organelle (GO: 0043228) were the dominant functional groups of BP, MF and CC, respectively. Further analysis of this gene subset to detect over-represented biological pathways identified 21 pathways with a greater significance (Table 4). For instance, genes involved in biological pathways such as cell cycle, DNA replication, endoreplication and alternative cell cycles, cell cycle checkpoints, pyrimidine metabolism and hormone biosynthesis were relatively highly expressed in somatic embryos than in WT_L.

**Table 4:** **Over-represented biological pathways detected within the more highly expressed gene subset identified in somatic embryos**

**Biological pathway**	**Probability**
Cell Cycle, Mitotic	1.10E-39
DNA Replication	2.50E-16
Endoreduplication and Alternative cell cycles	3.30E-13
Cell Cycle Checkpoints	9.30E-05
Pyrimidine metabolism (KEGG)	3.10E-04
Purine metabolism (KEGG)	1.90E-02
Cytokinins-O-glucoside biosynthesis (AraCyc)	2.50E-02
Cytokinins 7-N-glucoside biosynthesis (AraCyc)	2.50E-02
Cytokinins 9-N-glucoside biosynthesis (AraCyc)	2.50E-02
Anthocyanin biosynthesis (AraCyc)	2.60E-02
Sucrose Biosynthesis	2.80E-02
Methionine degradation II (AraCyc)	2.80E-02
G1/S DNA integrity checkpoint	2.80E-02
Glucosinolate biosynthesis from homomethionine (AraCyc)	4.10E-02
Diterpenoid biosynthesis (KEGG)	4.90E-02
de novo biosynthesis of purine nucleotides II (AraCyc)	5.40E-02
Glucosinolate biosynthesis from tryptophan (AraCyc)	6.30E-02
Glucosinolate biosynthesis from phenylalanine (AraCyc)	6.90E-02
Sucrose degradation to ethanol and lactate (anaerobic) (AraCyc)	7.70E-02
Sucrose biosynthesis (AraCyc)	8.30E-02
Gibberellin biosynthesis III (early C-13 hydroxylation) (AraCyc)	9.10E-02

The gene expression resources deposited in public repositories such as AtGenExpress (http://www.weigelworld.org/resources/microarray/AtGenExpress/), Genevestigator (https://www.genevestigator.com/gv/) and ArrayExpress (http://www.ebi.ac.uk/arrayexpress/) are useful to investigate expression patterns of gene(s)/gene families in variety of context i.e. expression across different tissue types, under diverse environmental conditions. We used Genevestigator expression datasets (microarray based) to further validate the expression patterns of a subset of those genes expressed more highly in somatic embryos than in leaf tissues by RNA-Seq. The resulting heat map for the embryogenic and leaf tissues revealed that the majority of genes selected are transcribed at a higher level in embryonic samples than in leaf tissues (Additional file 2).

Additionally, to identify the genes that encode for essential and non-redundant function, the subset of genes that were expressed at a higher level in somatic embryos were probed with the embryo defective mutant gene list reported in the SeedGenes database (http://www.seedgenes.org/). This database provides information on 481 essential genes and 888 mutants. It was evident that 101 (21%) of those genes are expressed at a higher level in our somatic embryogenic samples than in leaf samples (Additional file 3). These genes represent approximately 2% of the more highly expressed gene list detected in somatic embryos. Gene expression patterns based on microarray data (Genevestigator) also confirmed that the majority of these genes were expressed in reproductive tissues rather than in vegetative tissues (Additional file 2). On the other hand, our study detected 2,149 genes that were expressed at a higher level in leaf tissues (Log2 [FC] ≤ −2.0). Analysis of this gene subset identified 47 genes were present in the list of 481 embryo defective genes in the SeedGenes database. Further investigation of transcript levels using Genevestigator clearly showed that these 47 genes are expressed at a considerably higher level in leaf tissues than in embryogenic tissues (Additional file 4).

**Figure 8:**
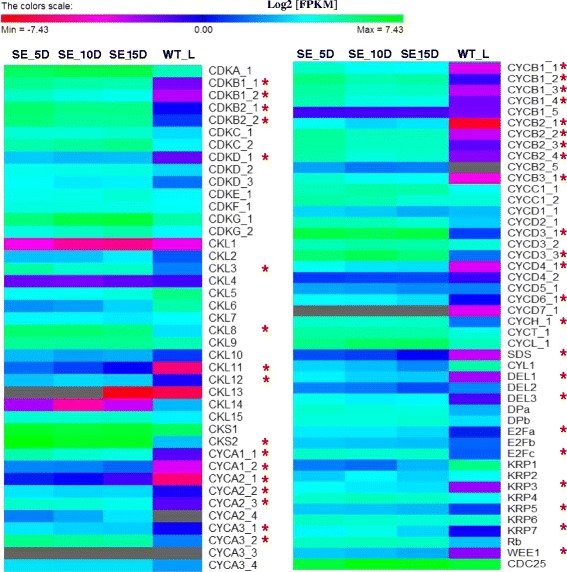
Transcript levels of core cell cycle related genes in embryogenic and leaf tissues. SE_5D, SE_10D and SE_15D are to represent embryogenic samples collected after 5, 10 and 15 d of *in vitro* culture, respectively. WT_L: WT leaf tissues. Ash coloured cells denote zero FPKM values (no transcripts detected). The DEGs detected between somatic embryo and leaf tissues are denoted by asterisks.

Establishment of *in vitro* embryogenic cultures requires activation of several specific gene subsets. Of these, genes that regulate the cell cycle and DNA replication play a key role in controlling active cell proliferation. In this study, comparative expression profile analysis of 88 core cell cycle genes [27,28] in somatic embryos and WT_L revealed that at least 39 genes are transcriptionally more active during SE with Log2 [FC] ≥ 2.0 (Figure 8; see Additional file 5 for the expression patterns of these genes based on microarray data from Genevestigator). Of these, five cyclin dependent kinases (CDKs) (*CDKB1;1*, *CDKB1;2*, *CDKB2;1*, *CDKB2;2* and *CDKD;1*), seven type-A cyclins (*CYCA1;1*, *CYCA1;2*, *CYCA2;1*, *CYCA2;2*, *CYCA2;3*, *CYCA3;1* and *CYCA3;2*), nine type-B cyclins (*CYCB1;1*, *CYCB1;2*, *CYCB1;3*, *CYCB1;4*, *CYCB2;1*, *CYCB2;2*, *CYCB2;3*, *CYCB2;4* and *CYCB3;1*) and four cyclin-D (*CYCD3;1*, *CYCD3;3*, *CYCD4;1* and *CYCD6;1*) were identified. Additionally, several *CDK*-*like* (*CKL*) genes (*CKL3*, *CKL8*, *CKL11*, and *CKL12*) and gene members of the E2F TFs that function in cyclin D/retinoblastoma/E2F pathway i.e. *E2Fa*, *E2Fc* and *E2L2* also showed noticeable accumulation of transcripts in embryogenic tissues than in WT leaf tissues. Several studies have reported involvement of key regulators of cell cycle during embryogenesis. For instance, expression analysis of a *CDK* in coconut SE has shown a gradual decrease in expression of *CnCDKA* during the course of somatic embryo development [29]. Moreover, over-expression of *Arabidopsis CDKA;1* caused developmental defects in embryos and lead to embryo lethality when it is knocked out [30,31]. It has been hypothesized that cell cycle genes may closely interact with hormone and other developmental signalling pathways to regulate *in vitro* embryogenesis [32]. However, additional research is needed to support this conclusion. Therefore, the expression patterns presented here may be useful to inform future research to investigate the molecular function of these genes during SE.

**Figure 9:**
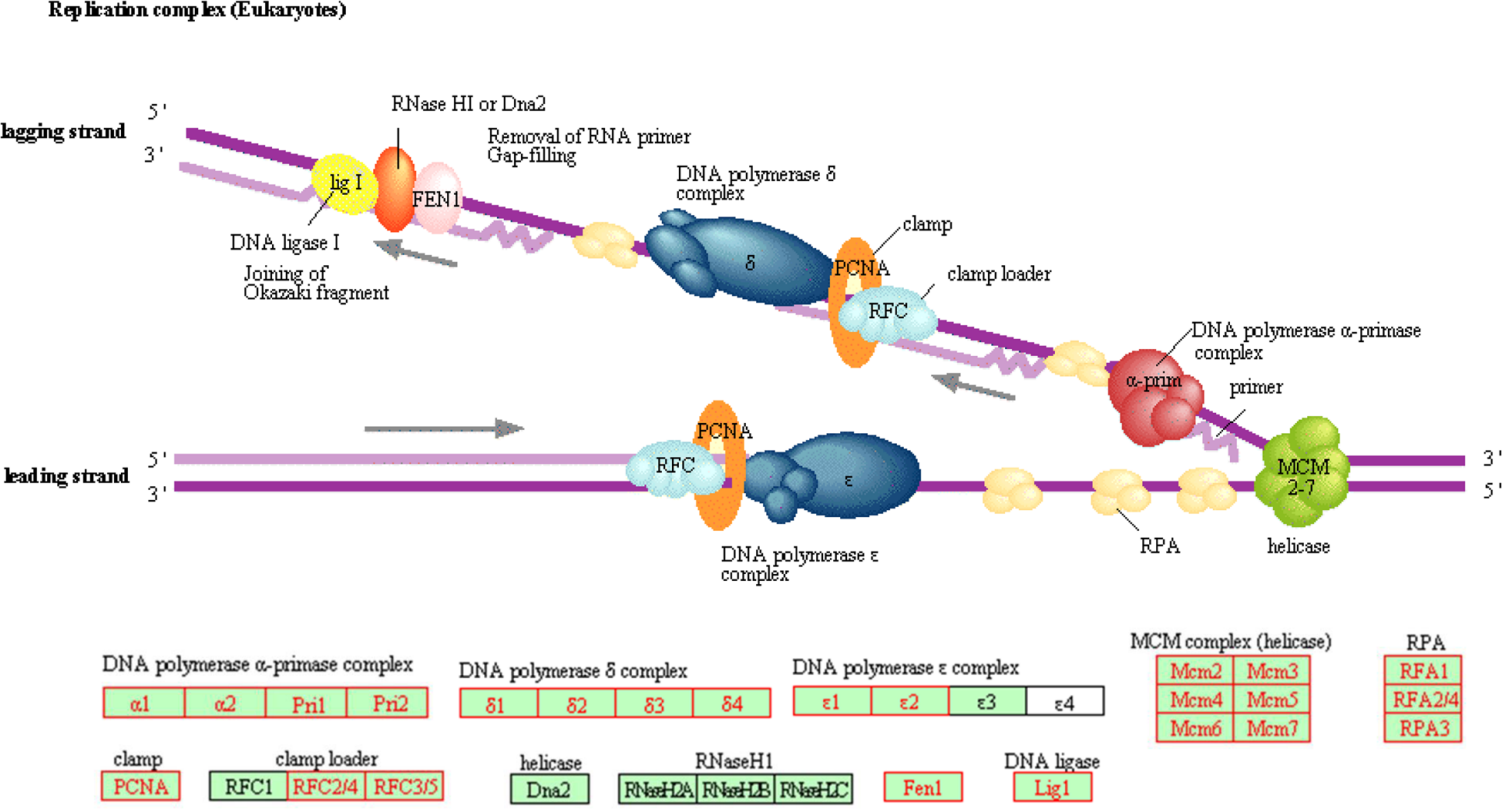
Transcriptionally more active genes that were mapped to the KEGG DNA replication machinery pathway. The genes that were more highly expressed in somatic embryos are highlighted in red boxes.

**Figure 10:**
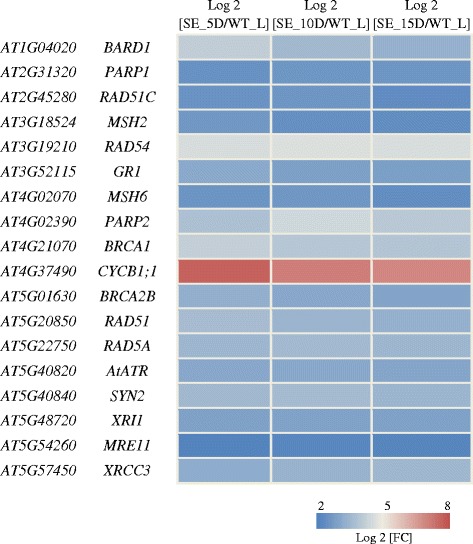
Differential expression of genes related to DSB repair mechanism between embryogenic and leaf tissues. SE_5D, SE_10D and SE_15D are to represent embryogenic samples collected after 5, 10 and 15 d of *in vitro* culture, respectively. WT_L: WT leaf tissues.

DNA replication plays a central role in the life cycle of plants. Mutations in key genes involved in this process have caused several embryo defective phenotypes in *Arabidopsis*. For instance, mutations in the catalytic subunit of the DNA polymerase epsilon complex have shown developmental defects during embryogenesis [33]. Therefore, to identify transcriptionally more active DNA replication related genes in embryogenic tissues, transcript levels of 65 genes related to DNA replication machinery [34] were filtered from embryonic and leaf transcriptomes, and compared. It was found that among these genes, 48 are transcriptionally more active in somatic embryos with Log2 [FC] ≥ 2.0 (see Additional file 6 for the expression patterns of these genes based on microarray data from Genevestigator); 30 of these genes were successfully mapped to the DNA replication pathway provided by the Kyoto Encyclopedia of Genes and Genomes (KEGG) (Figure 9). This is in accordance with the finding of Masuda et al. [35], who reported higher expression of *Arabidopsis* pre-replication complex components (i.e. *ORC1*-*6*, *CDT1a* and *CDC6a*) in reproductive tissues than in vegetative tissues. Further analysis of transcript levels for 32 genes related to DNA double strand break (DSB) repair mechanism identified 18 significantly expressed genes in somatic embryos (Log2 [FC] ≥ 2.0) (Figure 10). This included several well-studied DSB repair genes such as *GAMMA RESPONSE 1* (*GR1*), *RAS ASSOCIATED WITH DIABETES 51* (*RAD51*), *BREAST CANCER SUSCEPTIBLITY 1* (*BRCA1*), *MEIOTIC RECOMBINATION 11* (*MRE11*) and *KU70.* These finding collectively suggest the importance to DNA replication and repair mechanism during SE which involves active cell proliferation.

Several TF encoding genes (≈785) were also differentially expressed between two tissue types examined here. Of these, 342 genes were expressed more highly (Log2 ≥ 2.0) in all three somatic embryo samples than in leaf tissues. These genes belong to more than 40 different TF families including C2H2 (40 genes), AP2-EREBP (39), bHLH (31), MYB (26), homeobox (22), WRKY (22), bZIP (13) and MADS-box (12). Several of these TF encoding genes that are important in SE are listed in Table 5. The majority of stress responsive genes that are involved in ethylene responses have been classified in the TF family, AP2-EREBP. In our study we noted that at least 39 genes related to the AP2-EREBP family are more highly expressed in somatic embryos than in leaf tissues. Among those genes, both ethylene responsive TFs, *RAP2.6* (*AT1G43160*) and *RAP2.6 L* (*AT5G13330*) were detected at a noticeably higher level in somatic embryos (Log2 [FC] ≈ 6). These two specific TFs play an important role during stress signalling [36]. Additionally, several genes encoding for CYTOKININ RESPONSE FACTORS (CRFs) were also transcriptionally more active in somatic embryos than in leaf tissues i.e. *CRF1*, *CRF2*, *CRF3*, *CFR4*, *CFR5*, *CRF10* and *CRF11*. These AP2-domain containing TFs, together with type-B *Arabidopsis* response regulators, are involved in regulation of embryo, cotyledon and leaf development [37]. Significant expression of *RAP2.6*, *RAP2.6 L* and four *CRF*s (*CRF2*, *CRF3*, *CRF4* and *CRF5*) in embryogenic cultures has been reported in a qRT-PCR expression analysis of TFs [38].

**Table 5:** **Important TF genes that were more highly expressed in somatic embryos as compared to leaf tissues, ranked in order of TF family**

**Gene ID**	**Gene short name**	**TF family**	**Description**
*AT3G26790*	*FUS3*	ABI3VP1	Regulator of gene expression during late embryogenesis
*AT1G28300*	*LEC2*	ABI3VP1	
*AT3G24650*	*ABI3*	ABI3VP1	Essential for seed maturation and a central regulator in ABA signalling
*AT4G37750*	*ANT*	AP2-EREBP	Required for control of cell proliferation
*AT5G57390*	*AIL5*	AP2-EREBP	Essential for the developmental transition between the embryonic and vegetative phases
*AT5G17430*	*BBM*	AP2-EREBP	Similar to *AINTEGUMENTA* expressed in embryos
*AT5G64750*	*ABR1*	AP2-EREBP	Involved in ABA signalling
*AT4G11140*	*CRF1*	AP2-EREBP	Members of ERF subfamily; cytokinin response factors function redundantly to regulate the development of embryos, cotyledons and leaves
*AT1G68550*	*CRF10*	AP2-EREBP	
*AT3G25890*	*CRF11*	AP2-EREBP	
*AT4G23750*	*CRF2*	AP2-EREBP	
*AT5G53290*	*CRF3*	AP2-EREBP	
*AT4G27950*	*CRF4*	AP2-EREBP	
*AT2G46310*	*CRF5*	AP2-EREBP	
*AT3G20840*	*PLT1*	AP2-EREBP	A key effector for establishment of the stem cell niche during embryonic pattern formation
*AT1G51190*	*PLT2*	AP2-EREBP	
*AT1G19850*	*MP*	ARF	TF (IAA24) mediating embryo axis formation and vascular development
*AT2G41070*	*EEL*	bZIP	Homologous to ABI5; Located in the nucleus and expressed during seed maturation in the cotyledons and later in the whole embryo
*AT2G36270*	*ABI5*	bZIP	Regulates a subset of LEA genes; involved in ABA signalling during seed maturation and germination
*AT3G54810*	*GATA8*	C2C2-Gata	Expressed in the embryo axis and involved in germination
*AT3G50870*	*GATA18*	C2C2-Gata	A transcriptional regulator required to position the proembryo boundary in the early embryo
*AT5G07500*	*PEI1*	C2H2	An embryo-specific zinc finger TF required for heart-stage embryo formation
*AT5G47670*	*L1L*	CCAAT-HAP3	A regulator of embryo development
*AT1G62360*	*STM*	HOMEOBOX	Required for shoot apical meristem (SAM) formation during embryogenesis and for SAM function throughout the lifetime of the plant
*AT5G13790*	*AGL15*	MADS	Preferentially expressed during embryogenesis and key regulator of embryogenesis
*AT5G53950*	*CUC2*	NAC	With CUC1 redundantly required for embryonic apical meristem formation and cotyledon separation
*AT3G15170*	*CUC1*	NAC	Involved in shoot apical meristem formation and auxin-mediated lateral root formation
*AT1G66600*	*ABO3*	WRKY	Involved in ABA signalling

**Figure 11:**
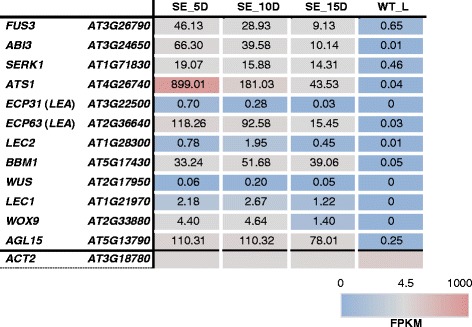
Transcript abundance for 12 somatic embryo marker genes in embryogenic and leaf tissues. SE_5D, SE_10D and SE_15D are to represent embryogenic samples collected after 5, 10 and 15 d of *in vitro* culture, respectively. WT_L: WT leaf tissues. *ACTIN 2* (*ACT2*) was used as the reference gene.

Studies so far have discovered several marker genes that regulate somatic-to-embryogenic cell fate in plants [5,39-43]. In order to further validate the expression levels of some of these genes, the transcript levels of 12 well-studied marker genes were filtered from our transcriptome data. As expected none of the marker genes selected is expressed at a high level in leaf tissues (Figure 11). It was evident that *FUSCA 3* (*FUS3*), *ABA INSENSITIVE 3* (*ABI3*), *SOMATIC EMBRYOGENESIS RECEPTOR KINASE 1* (*SERK1*), *ATS1* and the *LEA* genes i.e. *ECP31* and *ECP63* are expressed at a higher level during early SE, with a gradual reduction during subsequent embryo maturation. Additionally, *LEAFY COTYLEDON 1* (*LEC1*), *WUSCHEL RELATED HOMEOBOX 9* (*WOX9*) and *AGAMOUS-like 15* (*AGL15*) genes also showed accumulation of more transcripts in early stages as compared to SE_15D; however, the transcripts levels remained relatively stable during 5 and 10 d after *in vitro* embryogenesis. Considerable expression of *LEAFY COTYLEDON 2* (*LEC2*), *BABY BOOM* (*BBM1*) and *WUSCHEL* (*WUS*) genes was noted in embryos after 10 d of *in vitro* culture. Interestingly, the two embryo specific genes, *ATS1* and *ATS3* [44] showed a similar pattern of transcript accumulation during *in vitro* embryogenesis where the expression was markedly high in embryos after 5 d of *in vitro* culture and then reduced as they develop. In addition, transcripts of both genes were noticeably more abundant in somatic embryos than in WT leaf tissues. Although it has been hypothesized that both of these embryo specific genes may function in maintaining subcellular embryogenic structures and establishing storage material within the cell [44], experimental validation has not yet been reported. Based on our transcript information, it is more likely that both *ATS1* and *ATS3* genes may have a regulatory role during early SE.

### Expression patterns of genes related to epigenetic modifications

SE is a highly dynamic developmental process that involves active dedifferentiation of somatic cells, followed by the induction and maturation of embryos. The process of induction of *in vitro* embryos is mainly regulated by plant growth regulators and stress responses. Apart from that, these factors may contribute to induce epigenetic modifications during SE. Therefore, to gain insight into the expression patterns of genes that regulate these epigenetic signatures, transcript levels of genes related to DNA methylation/demethylation and histone acetylation/deacetylation were studied in-depth. This would provide clues about the level of methylation during *in vitro* embryogenesis.

**Figure 12:**
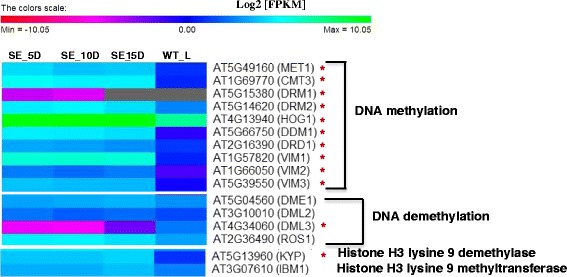
Transcript levels of genes related DNA methylation/demethylation in embryogenic and leaf tissues. SE_5D, SE_10D and SE_15D are to represent embryogenic samples collected after 5, 10 and 15 d of *in vitro* culture, respectively. WT_L: WT leaf tissues. Ash coloured cells denote zero FPKM values (no transcripts detected). The DEGs detected between somatic embryo and leaf tissues are denoted by asterisks.

Cytosine DNA methylation is one of the well-studied epigenetic modifications in higher plants and is involved in regulation of gene expression and silencing of TEs [45]. In addition, it controls various processes such as embryogenesis and genomic imprinting [46]. This process involves incorporation of a methyl group to the 5′ position of the cytosine base by a group of enzymes called DNA methyltransferases. Although there was no considerable variation in transcript levels of genes related to DNA methylation/demethylation during SE, a marked difference was noticed for these genes between somatic embryo and WT_L transcriptomes (Figure 12; see Additional file 7 for the expression patterns of these genes based on microarray data from Genevestigator). Both *METHYLTRANSFERASE 1* (*MET1*) and *CHROMOMETHYLASE 3* (*CMT3*) genes that play a key role in maintaining cytosine DNA methylation during *Arabidopsis* embryogenesis [47] were transcriptionally more active in embryogenic tissues (Log2 [FC] ≥ 2.0). In addition, *DOMAINS REARRANGED METHYLTRANSFERASE 2* (*DRM2*), the main *de novo* methyltransferase found in *Arabidopsis* [48] also showed accumulation of more transcripts in somatic embryos. The single gene mutants of *MET1* (*met1-6*) in *Arabidopsis* display developmental defects in ZEs i.e. abnormal cell divisions during early embryogenesis and delayed development as compared to WT embryos [47]. In addition, the reduced expression levels observed for genes that specify cell fate during early embryogenesis (*WUSCHEL RELATED HOMEOBOX 2* (*WOX2*), *WOX8* and *YODA* (*YDA*)) in *met1-6* mutants highlights the importance of DNA methylation in maintaining cell identify during early embryogenesis [47]. Likewise, the double mutants of *MET1* and *CMT3* genes have reduced seed viability. Although further evidence is required, it is believed that DNA methylation may be directly or indirectly involved in the regulation of polar auxin transport during early embryogenesis [47]. Thus, it is possible that the increased expression levels observed for most of the genes related to cytosine DNA methylation in somatic embryos may link to hormone and developmental signalling pathways that take place during *in vitro* embryogenesis.

Similar to DNA methylation related genes, the histone methylation related gene, *KRYTONITE* (*KYP*) also showed higher expression in somatic embryos (Figure 12). This histone methyltransferase, *KYP* functions together with *CMT3* to induce and maintain CHG trinucleotide methylation in TEs [46,49]. Increased accumulation of both *CMT3* and *KYP* transcripts in somatic embryos than in leaf tissues (Log2 [FC] ≥ 2.0) suggests activation of *CMT3* – *KYP* mediated cytosine methylation during SE to inactivate mobile transposons.

Furthermore, the proteins that bind to methylated cytosine also play a key crucial role in maintaining epigenetic status. Of these, three VARIANT IN METHYLATION (VIM) proteins have been identified as predominant regulators that maintain global CG dinucleotide methylation patterns and gene silencing in *Arabidopsis* [50]. Expression analysis of VIM encoding genes through RT-PCR reports higher transcript levels for *VIM1* and *VIM2* in *Arabidopsis* inflorescences than in leaves. Moreover, a relatively similar level of gene expression has been detected in both inflorescences and leaves for *VIM3*. However, we found that all three VIM encoding genes are expressed at a considerably higher level in somatic embryos than in leaf tissues (Log2 [FC] ≥ 2.0). Of these, *VIM1* showed the highest transcript abundance in embryogenic tissues; this suggests *VIM*s may play a role in maintaining methylation patterns during embryogenesis *in vitro*.

In addition to DNA methylation, DNA demethylation is also equally important in regulation of gene expression patterns during developmental processes. In *Arabidopsis*, three genes related to the DME family (*DEMETER 1* (*DME1*), *DEMETER-LIKE 2* (*DML2*) and *DEMETER-LIKE 3* (*DML3*)) and a homolog of DME known as *REPRESSOR OF SILENCING 1* (*ROS1*), that contain a DNA glycosylase domain, are involved in DNA demethylation [46]. It was evident that with the exception of *DML3*, other genes related to DNA demethylation were expressed at a relatively similar level in somatic embryos and WT leaf tissues. However, *DML3* that catalyses active removal of 5-methylcytosine showed considerably lower expression in somatic embryos as compared to WT_L with an average Log2 ratio of −6. It is reported that although *DML3* is expressed in siliques and leaves, it is not expressed in mature seeds. In addition, the mRNA stability of *DML3* may depend on the plant tissue type [51]. Thus, based on our expression data, it can be assumed that *DML3* transcripts are more stable in leaves as compared to embryos.

Histone acetylation/deacetylation is another important post translational histone modification that regulates gene expression patterns during plant development as well as in response to various environmental conditions [52]. In general, hyperacetylation activates transcription and mediated by a group of enzymes known as histone acetyltransferases (HATs). The present study found that with the exception of the CBP family member *HAC2*, other *HAT*s are expressed stably during SE. The *HAC2* gene was expressed at a considerably higher level in later stages (SE_10D and SE_15D) as compared to SE_5D. In addition, the differential gene expression analysis of HATs between embryogenic and leaf tissues showed noticeable accumulation of *HAC2* transcript in somatic embryos than in WT_L. However, to date no histone acetylation activity has been reported for this gene in *Arabidopsis* [53]. In animals, CBP family proteins are considered as active transcriptional co-activators that regulate processes such as cell cycle and cell differentiation. Moreover, CBP family members have been identified as essential regulators of mouse embryogenesis and pattern formation [54]. Therefore, detection of increased expression of *HAC2* gene in somatic embryos suggests that it is likely to function during SE, which also involves active cell divisions. In addition to *HAC2*, two genes from the GNAT family, *HAG2* and *HAG3* also showed accumulation of more transcripts in somatic embryos than in leaf tissues with an average Log2 [FC] of 2.0. However, the majority of plant HATs have been identified by sequence characterization to other eukaryotes; thus, the functional characterization of the majority of these genes remains largely unknown. Some of the functionally characterized *HAT*s include *HAG1*, *HAF2*, *HAC1*, *HAC5* and *HAC12* [55]. For instance, a study on plant *HAC1* has shown its function in reproductive and vegetative development in *Arabidopsis* [56]; T-DNA mutants of *HAC1* have shown reduced fertility with shorter siliques as compared to WT plants and late flowering phenotypes [57]. Therefore, it is possible that *HAG2*, *HAG3* and *HAC2* may also have a function in embryogenesis.

On the other hand, histone deacetylation/hypoacetylation may lead to a transcriptional repression. This process is catalysed by histone deacetylases (HDACs) [45,58]. Expression profile analysis of 18 *HDAC*s comprising 12 from the RPD3 family, four from the plant specific HD-tuins family and two from the Sirtuin HDAC family showed that with the exception of *HDA7*, all the *HDAC*s are expressed throughout the SE in a relatively stable manner. Transcripts for the *HDA7* gene were only detected in SE_10D. Interestingly, it was noted that all four HD-tuins family members expressed at a relatively similar level in somatic embryos and shared a similar expression pattern during *in vitro* embryo development. A similar pattern of gene expression has been observed for HD-tuins family members in somatic embryos and seed derived embryos through *in situ* hybridization, indicating a functional redundancy of those genes in plant development [58]. Additionally, these genes were transcriptionally more active in embryogenic tissues than in leaf tissues. Similarly, Zhou et al. [59] report accumulation of *HDT1*, *HDT2* and *HDT3* transcripts in somatic embryos derived from transgenic *Arabidopsis* lines that over express *BBM* gene. However, it was not clear, whether the accumulation of HD-tuins type genes is due to formation of somatic embryos or over-expression of *BBM* in transgenic lines that exhibit a higher frequency of induced embryos [59]. The expression information in the present study provides strong support for the suggestion that HD-tuins family HDACs are important in *Arabidopsis* SE and are more likely to regulate histone modifications in target genes to maintain a high methylation status during SE. However, additional information is needed to determine whether the increased transcript levels observed for HD-tuins in somatic embryos has a link to hormone or developmental signalling pathways that take place during embryogenesis.

In general, detection of increased transcript levels for key genes involved in DNA methylation and histone deacetylation provides an indirect indication of increased epigenetic modifications during *in vitro* embryogenesis. However, it is not clear whether the expression changes we observed are due to *in vitro* conditions (i.e. externally supplied auxin, stress responses) or increased epigenetic signatures. Therefore, it would be valuable to conduct a global analysis of the epigenome architecture of somatic embryos in order to understand the expression patterns of diverse embryogenesis related genes.

### *In silico* expression analysis to identify genes more likely to be involved in SE

Identification of genes more likely to be involved in somatic embryo development would help in the discovery of novel molecular markers for future embryogenesis studies. As a preliminary study, to obtain an estimate of the overlap between genes expressed in somatic embryos and actively dividing callus, 100 genes were randomly selected from our list of genes that showed a higher level of expression in somatic embryos than in non-dividing leaf tissues (a total of (4,951)). It was considered likely that the majority of genes highly expressed in somatic embryos could also be expressed at a higher level in actively dividing cells, for example in callus. In order to address this possibility a comparison of expression data from somatic embryos, callus and leaf was conducted. This concluded that approximately 5% of genes are expressed at a higher level in somatic embryos than in actively dividing callus or non-dividing leaf tissues (Log2 [FC] ≥ 2.0) (See Additional file 8 for the clustering analysis of microarray expression data from somatic embryos (original repository: Gene Expression Omnibus (GEO (GSE17610); experiment ID: AT-00508), callus (original repository: GEO (GSE8994); experiment ID: AT-00265) and leaf tissues (original repository: GEO (GSE8994); experiment ID: AT-00265). These data were filtered for the randomly selected gene list (100 genes)).

**Figure 13:**
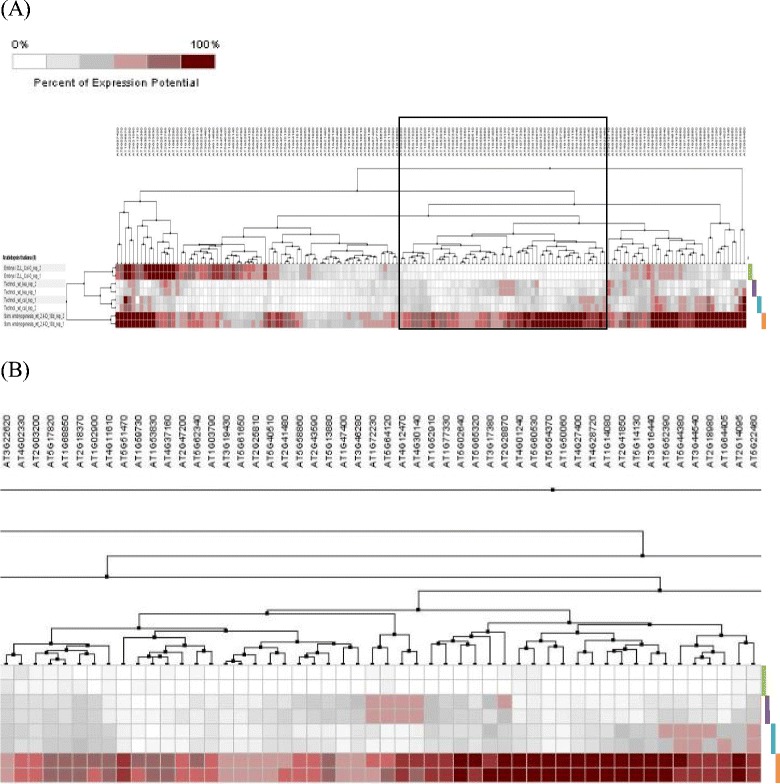
Hierarchical clustering (using Euclidian distance measure) for a list of 158 genes expressed at a higher level in somatic embryos (AT-00508) than in callus (AT-00265), torpedo stage zygotic embryos (AT-00629) or leaf tissues (AT-00265). **(A)** Hierarchical clustering of 158 genes; **(B)** Expansion of the clade that showed a higher level of expression in somatic embryos than in callus, torpedo stage embryos or leaf tissues. Green, purple, blue and orange lines represent expression data for torpedo stage zygotic embryos, leaf tissues, callus and somatic embryos, respectively. The Genevestigator hierarchical clustering tool was used to construct the clustering tree.

**Figure 14:**
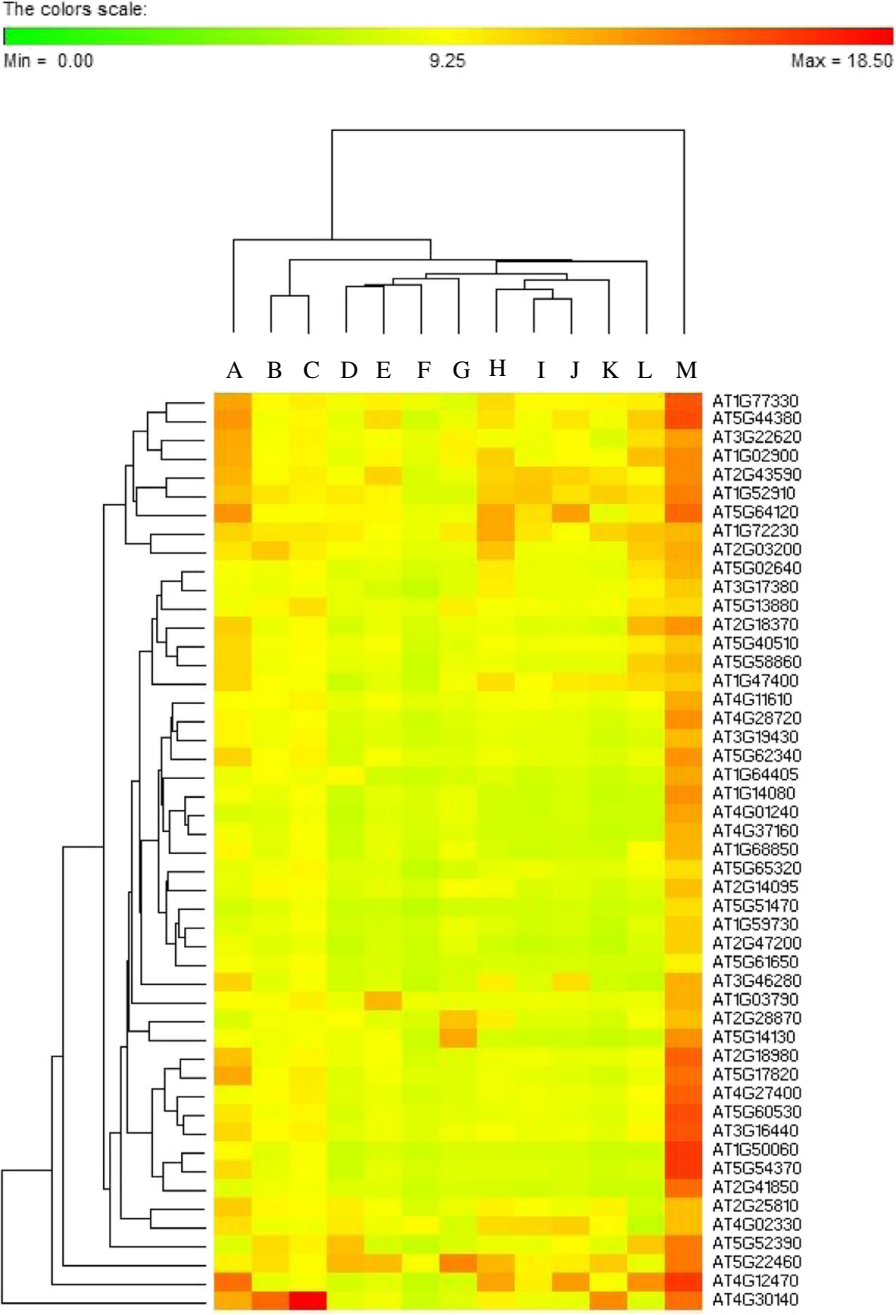
Expression pattern 49 genes expressed at a higher level in somatic embryos than in callus, torpedo stage zygotic embryos or leaf tissues in a range of anatomical parts. A: seedling; B: stamen; C: pollen; D: pistil; E: seed; F: embryo; G: testa; H: inflorescence stem; I: juvenile leaf; J: adult leaf; K: shoot apex; L: hypocotyl; M: somatic embryos. Expression data represent average log 2 signal values for replicates of each tissue sample.

In summary, comparison of microarray gene expression data for somatic embryos (AT-00508) and callus (AT-00265) tissues identified a minimum set of 158 genes that are more highly expressed in somatic embryos than in actively dividing callus cells. Furthermore, subsequent hierarchical clustering of expression data for these 158 genes in samples: somatic embryos (AT-00508), callus (AT-00265), leaf (AT-00265) and torpedo stage zygotic embryos (original repository: GEO (GSE47884); experiment ID: AT-00629) exhibited a distinct cluster with 49 genes (Figure 13) having a comparatively higher level of expression in somatic embryos than in torpedo stage zygotic embryos (See Additional file 9 for the gene description). Furthermore, expression analysis of these genes in other anatomical parts using the Genevestigator tool showed the majority of these genes have a distinct pattern of expression in somatic embryos as compared to other anatomical parts compared (Figure 14). Therefore, these genes could be considered as potential candidate genes for future *in vitro* embryogenesis related studies.

Interestingly, this subset of 49 genes included several genes responsible for oxidative stress such as *AT1G68850*, *AT2G18980*, *AT5G14130*, *AT5G17820* and *AT5G44380*; genes responsible for salt stress, *AT4G02330* and *AT4G27400*; genes encoding for LEA proteins, AT3G19430, AT4G27400, AT5G54370 and AT5G60530; genes involved in auxin biosynthesis, *AT4G28720* and *AT5G51470*. Moreover, further analysis of these genes revealed the four genes encoding for LEA proteins are positively co-expressed (Pearson’s correlation coefficient > 0.60) with both the auxin biosynthesis genes, *AT4G28720* and *AT5G51470* (Additional file 10). Although these genes were expressed at a higher level in somatic embryos than in other tissues, further studies are recommended to speculate whether these genes are differentially expressed during SE or in response to exogenously supplied auxin, 2, 4-dichlorophenoxyacetic acid (2, 4-D).

## Conclusions

In summary, this study provides a high resolution transcriptome dataset for *Arabidopsis* SE. A comparative analysis of expression profiles between different somatic embryo developmental time-points as well as between embryogenic and leaf tissues provided subsets of DEGs. Functional characterization of those gene subsets based on significantly enriched GO terms and over-represented biological pathways is presented here. However, further functional characterization and validation of these genes at a molecular level is recommended. Interestingly, the comparison of somatic embryo and leaf tissue derived transcriptomes revealed distinct expression profiles for the majority of genes involved in cell cycle, DNA replication and repair mechanism, DNA cytosine methylation and histone deacetylation. The latter results suggest a particular role for epigenetic regulation in the SE process, and may provide useful leads in the design of systems for the improved induction of embryonic cells from somatic tissue. Interestingly, *in silico* expression analysis of genes using publicly available microarray expression data identified a subset of 49 genes that are more likely to be involved in SE, and could be considered as potential candidate genes for future embryogenesis studies *in vitro*. In conclusion, the transcriptomic data generated in the present study for *Arabidopsis* somatic embryos provides insights into future functional studies and into the development of gene regulatory networks for model species, as a means of understanding the molecular mechanisms that control SE.

## Methods

### Plant material and growth conditions

Seeds of the *A. thaliana* Columbia ecotype (accession number: N70000) were supplied by the Nottingham *Arabidopsis* Stock Centre, UK. Mother plants were grown on vermiculite containing soil at 25°C in 60% relative humidity with a photoperiod of 16 h under controlled environmental conditions in Fitotron plant growth chambers (Weiss Gallenkamp, UK).

The direct somatic embryo induction method described by Gaj [60] was used to induce somatic embryos from WT *Arabidopsis*. In brief, immature zygotic embryos (IZEs), dissected from green siliques of 6-to-8 week old plants, containing embryos at the early bent-cotyledonary stage were used as explants. Initially, all the siliques were surface sterilized by a 10 min incubation in a commercial bleach (Domestos™) containing 20 drops of Tween-20® (BDH Laboratory supplies, UK) per 100 ml. After rinsing in sterile water for three times, IZEs (approximately 400–500 μm in size) were excised by opening surface sterilized siliques, under a dissecting microscope (Stemi SR, Zeiss, Germany), and used to induce somatic embryos. Ten IZEs were inoculated on each Petri dish (90 mm) containing approximately 20 ml of autoclaved basal Gamborg’s B5 medium (containing vitamins; Duchefa Biochemie, The Netherlands) supplemented with 20.0 g/L sucrose (BDH Laboratory supplies, UK), 3.5 g/L Phytagel (Sigma-Aldrich, USA), 5.0 μM of 2, 4-D (Sigma-Aldrich, USA) at pH 5.8 ± 0.01. The cultures were then incubated at 24°C with a photoperiod of 16 h, under white fluorescent light (90 μmol m^−2^ s^−1^ intensity).

### Tissue sampling and RNA extraction

For RNA extraction and subsequent transcriptome sequencing, somatic embryos were collected at three distinct time-points after initial inoculation, 5 d (SE_5D), 10 d (SE_10D) and 15 d (SE_15D) (Additional file 11). Approximately 700 somatic embryos induced at each time-point were pooled together to obtain a sufficient quantity of starting material for RNA extraction. In addition, two mature leaves collected at the time of flowering from WT plants (WT_L) were pooled to extract RNA. Total RNA was extracted using the RNeasy® Plant Mini Kit (Qiagen, UK) according to the manufacturer’s instructions and treated with DNase I (Qiagen, UK) to remove any contaminated DNA. The eluted total RNA was quantified using a BioAnalyzer 2100 (Agilent Technologies, CA) by Source Bioscience, UK.

### cDNA library construction and Illumina RNA-Seq

The cDNA libraries were constructed and sequenced by Source Bioscience, UK in accordance with the Illumina TruSeq RNA sample preparation guide v2 for Illumina paired-end multiplexed sequencing. In brief, the poly-A-mRNA in the extracted total RNA samples was purified using Illumina poly-T oligo-attached magnetic beads in two rounds of purification steps according to the manufacturer’s instruction. During the second step of poly-A RNA elution, the mRNA was fragmented and primed with random hexamers for cDNA synthesis. The first strand cDNA was synthesized from fragmented mRNA using reverse transcriptase and random primers. In a subsequent step, the RNA template was removed and a replacement was synthesized to construct double-stranded cDNA. After double stranded cDNA synthesis, ends were repaired and an A-base was added to the blunt end fragments. Thereafter, Illumina indexing adapters (Table 6) were ligated according to the standard protocol for pooling of samples prior to sequencing and for subsequent identification of pooled samples in downstream analysis. The cDNA fragments that have adapter molecules on both ends were subjected to 15 rounds of PCR amplification. The concentration and size distribution of the synthesized cDNA libraries were confirmed using an Agilent BioAnalyzer 2100. The successfully amplified and indexed libraries were pooled prior to sequencing (two samples per lane). The resulted pools were diluted to 10 nM. The molarity and size distribution were confirmed using an Agilent BioAnalyzer 2100. Finally, pooled samples were loaded at a concentration of 8 pM into each lane of an Illumina HiSeq 2000 flow cell v3 and sequenced with 50 bp paired-end reads.

**Table 6:** **True Seq adapter sequences**

**Sample ID**	**Adapter sequence (5′ - > 3′)**
SE_5D	GATCGGAAGAGCACACGTCTGAACTCCAGTCACATCACGATCTCGTATGCCGTCTTCTGCTTG
SE_10D	GATCGGAAGAGCACACGTCTGAACTCCAGTCACCGATGTATCTCGTATGCCGTCTTCTGCTTG
SE_15D	GATCGGAAGAGCACACGTCTGAACTCCAGTCACTTAGGCATCTCGTATGCCGTCTTCTGCTTG
WT_L	GATCGGAAGAGCACACGTCTGAACTCCAGTCACTGACCAATCTCGTATGCCGTCTTCTGCTTG

### Mapping sequence reads to the *Arabidopsis* reference genome

Pre-built TAIR10 index was downloaded from the Illumina iGenomes webpage (http://support.illumina.com/sequencing/sequencing_software/igenome.html; Arabidopsis_thaliana_Ensembl_TAIR10.tar.gz) and used as the reference for mapping. Quality of each raw sequence data file was analysed using open-source software FastQC (http://www.bioinformatics.babraham.ac.uk/projects/fastqc/). After removing adapter sequences, the high quality sequence reads were aligned and assembled to the *A. thaliana* reference genome (TAIR10) using TopHat 2.0.6 (http://ccb.jhu.edu/software/tophat/index.shtml) with Bowtie 2 (http://bowtie-bio.sourceforge.net/index.shtml) with the default parameters, allowing only 2 base mismatches per read alignment [61]. Aligned sequence reads were visualized using the IGV version 2.2 (http://www.broadinstitute.org/igv/) and Integrated Genome Browser version 7.0.4 (IGB) (http://bioviz.org/igb/). The general statistics of mapped reads were acquired using the Sequence Alignment/Map (SAM) tool (http://samtools.sourceforge.net/).

### Normalization of transcript levels of expressed genes

The assembled transcripts were merged with the *Arabidopsis* reference annotation downloaded from the Illumina iGenome (Arabidopsis_thaliana_Ensembl_TAIR10.tar.gz) and the transcript abundance were estimated in terms of FPKM.

### Analysis of differentially expressed genes (DEGs) and downstream bioinformatic analysis

The FC was calculated as a ratio of transcript levels (FPKM) between different somatic embryo developmental time-points or between different tissue types (embryogenic vs. leaf). Genes with −2.0 ≥ Log2 [FC] ≥ 2.0 were considered as DEGs. K-mean clustering on these gene subsets was performed using the MultiExperiment Viewer (MeV) software with 10 user defined clusters (http://www.tm4.org/).

The PLAZA web tool (http://bioinformatics.psb.ugent.be/plaza/) [62] was used to identify significantly enriched GO terms within a given subset of genes. In addition, functional networks based on enriched biological processes related GO terms were generated using Cytoscape (http://www.cytoscape.org/) software with the ClueGO plugin [63]. The SkyPainter tool in the *Arabidopsis* Reactome was used to identify the most common plant biological pathways (http://arabidopsisreactome.org/userguide/userguide.html) that exist within the subsets of DEGs.

Transcript levels of genes related to DNA replication and repair mechanisms [34], cell cycle [27,28], methylation/demethylation [46] and histone acetylation/deacetylation [57] were filtered from our transcriptome data to provide a comprehensive overview of their gene expression patterns during *in vitro* embryogenesis.

### *In silico* validation of gene expression patterns

To validate the gene expression patterns, Affymetrix microarray expression data were filtered for somatic embryos, zygotic embryos, callus and leaf tissues, and used to produce expression heat maps using the Genevestigator software tool (https://genevestigator.com/gv/plant.jsp).

### Availability of supporting data

The data set(s) supporting the results of this article are available in the European Bioinformatics Institute ArrayExpress repository, E-MTAB-2403 and E-MTAB-2465 (www.ebi.ac.uk/arrayexpress/).

## Additional files

Additional file 1:
**Transcript data of somatic embryos and leaf tissues.**
Additional file 2:
**Expression data extracted from the Genevestigator for a subset of highly expressed genes detected in somatic embryos than in leaf tissues.** (A) Mature leaf tissue (original repository: AtGenExpress (expression atlas of *Arabidopsis* development); (B) Different stages of seed development (original repository: ArrayExpress (E-GEOD-5634); (C) Somatic embryos after 10 d of culture (original repository: (GEO (GSE17610)). Additional file 3:
**List of potential candidate genes for molecular studies.** This subset of genes was prepared from a list of more highly expressed genes detected in somatic embryos in the present study which had links to known mutant seed phenotypes (SeedGenes database). Additional file 4:
**Expression data extracted from the Genevestigator for a subset of highly expressed genes detected in WT leaf than in somatic embryos.** (A) Mature leaf tissue (original repository: AtGenExpress (expression atlas of *Arabidopsis* development); (B) Different stages of seed development (original repository: ArrayExpress (E-GEOD-5634); (C) Somatic embryos after 10 d of culture (original repository: GEO (GSE17610)). Additional file 5:
**Expression data extracted from the Genevestigator for a subset of genes related to cell cycle.** (A) Mature leaf tissue (original repository: AtGenExpress (expression atlas of *Arabidopsis* development); (B) Different stages of seed development (original repository: ArrayExpress (E-GEOD-5634); (C) Somatic embryos after 10 d of culture (original repository: GEO (GSE17610)). Additional file 6:
**Expression data extracted from the Genevestigator for a subset of genes related to DNA replication.** (A) Mature leaf tissue (original repository: AtGenExpress (expression atlas of *Arabidopsis* development); (B) Different stages of seed development (original repository: ArrayExpress (E-GEOD-5634); (C) Somatic embryos after 10 d of culture (original repository: GEO (GSE17610)). Additional file 7:
**Expression data extracted from the Genevestigator for a subset of genes related to DNA cytosine methylation.** (A) Mature leaf tissue (original repository: AtGenExpress (expression atlas of *Arabidopsis* development); (B) Different stages of seed development (original repository: ArrayExpress (E-GEOD-5634); (C) Somatic embryos after 10 d of culture (original repository: GEO (GSE17610)). Additional file 8:
**Hierarchical clustering (using Euclidian distance measure) for a subset of 100 randomly selected genes, using microarray expression data from somatic embryos (AT-00508), callus (AT-00265) and leaf (AT-00265) in** ***Arabidopsis.*** The gene list employed in this study was selected from the list of more highly expressed genes identified in somatic embryos in the present study. Genevestigator hierarchical clustering tool was used to construct the clustering tree. The genes more highly expressed in somatic embryos as compared to leaf and callus tissues are highlighted in yellow (Log2 [FC] ≥ 2.0). Orange, blue and purple colours highlight the samples from somatic embryos, callus and leaf tissues, respectively. Additional file 9:
**Description of the 49 genes that exhibited a higher level of expression in somatic embryos (AT-00508) than in callus (AT-00265), torpedo stage zygotic embryos (AT-00629) or leaf tissues (AT-00265), based on microarray data extracted from the Genevestigator tool.**
Additional file 10:
**List of the top two hundred genes most highly co-expressed with** ***AT4G28720*** **and** ***AT5G51470.*** Co-expression data generated using the Genevestigator co-expression analysis tool, and based on 72 different tissue samples. Additional file 11:
**Time-points selected for tissue sampling and total RNA extraction from IZE explants cultured on B5 medium supplemented with 5 μM 2, 4-D.** Scale bars = 200 μm.

## Abbreviations

2,4-D: 2, 4-dichlorophenoxyacetic acid
bHLH: Basic helix-loop-helix
BP: Biological processes
CC: Cellular components
DEG: Differentially expressed gene
FC: Fold change
FPKM: Fragments Per Kilobase of exon per Million fragments mapped
GEO: Gene Expression Omnibus
GO: Gene Ontology
IZE: Immature zygotic embryo
MF: Molecular function
RNA-Seq: RNA sequencing
SE: Somatic embryogenesis
SE_5D: Transcriptome of embryogenic cultures after 5 d of inoculation
SE_10D: Transcriptome of embryogenic cultures after 10 d of inoculation
SE_15D: Transcriptome of embryogenic cultures after 15 d of inoculation
TE: Transposable element
TF: Transcription factor
WT: Wild type
WT_L: Transcriptome of WT leaf tissues
ZE: Zygotic embryo
