# Supplementary figures and images for: Global scale transcriptome analysis of Arabidopsis embryogenesis in vitro
Source: BMC Genomics. 2015 Apr 16;16(1):301. doi: 10.1186/s12864-015-1504-6 (PMC4404573; doi:10.1186/s12864-015-1504-6)

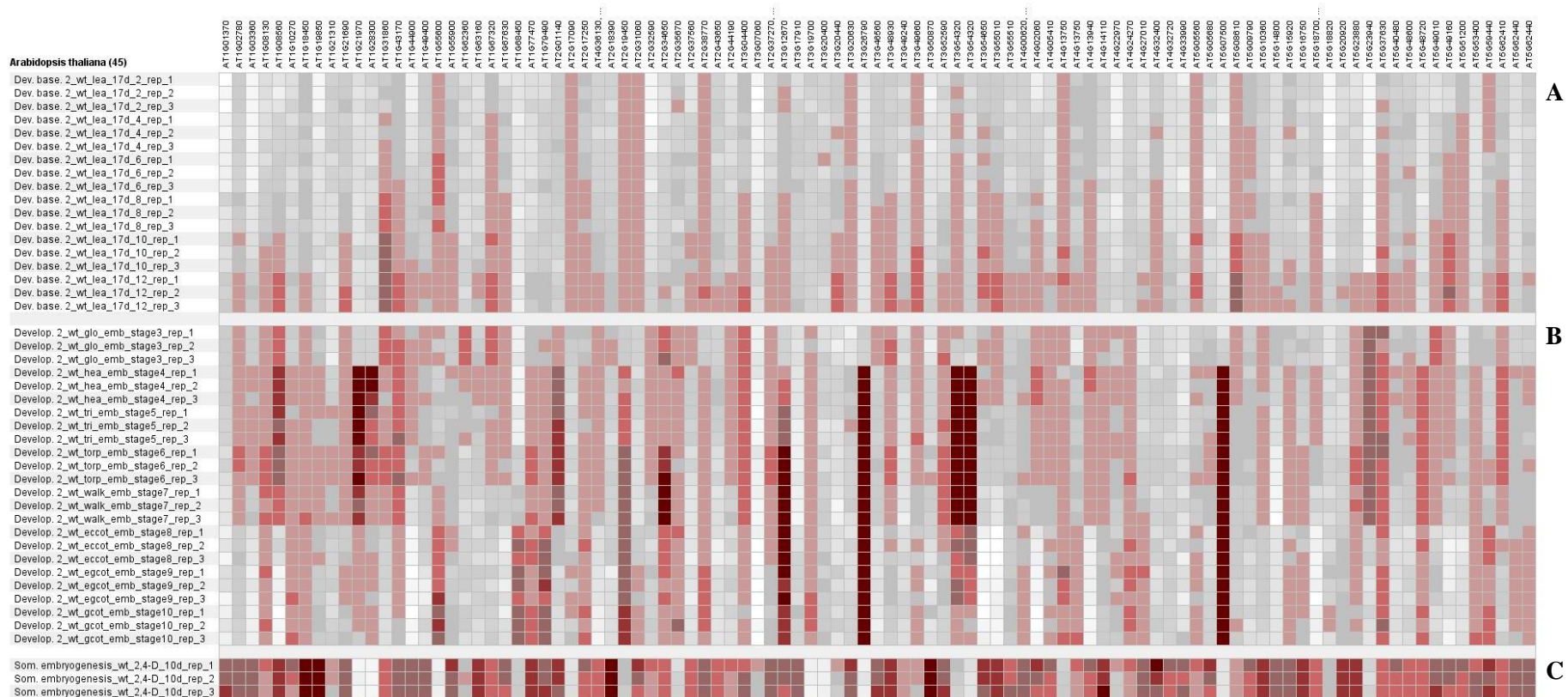

Supplement: Additional file 2: — Expression data extracted from the Genevestigator for a subset of highly expressed genes detected in somatic embryos than in leaf tissues. (A) Mature leaf tissue (original repository: AtGenExpress (expression atlas of Arabidopsis development); (B) Different stages of seed development (original repository: ArrayExpress (E-GEOD-5634); (C) Somatic embryos after 10 d of culture (original repository: (GEO (GSE17610)). [file 12864_2015_1504_MOESM2_ESM.pdf]

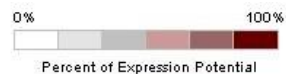

# **Arabidopsis thaliana (45)**

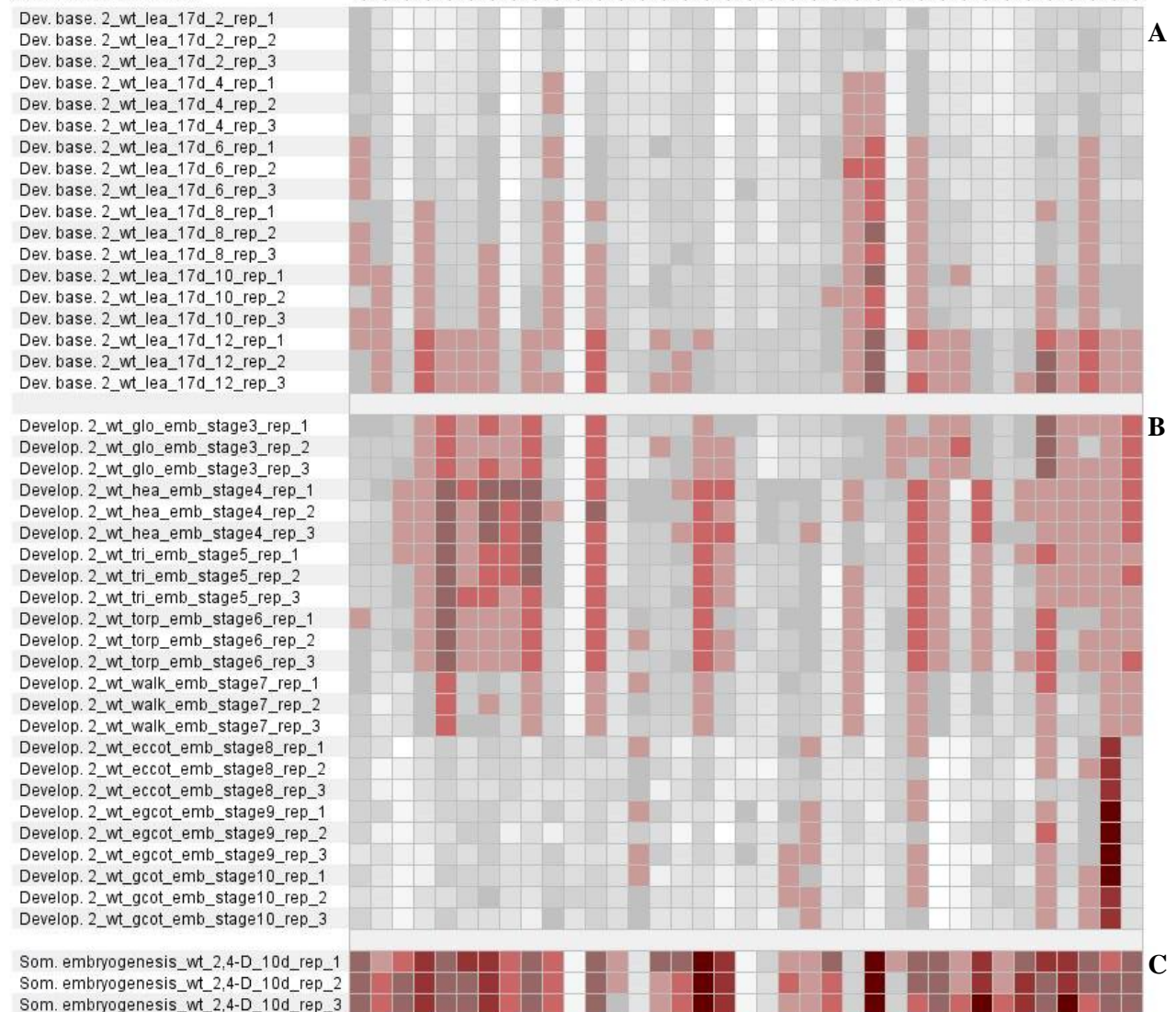

Supplement: Additional file 5: — Expression data extracted from the Genevestigator for a subset of genes related to cell cycle. (A) Mature leaf tissue (original repository: AtGenExpress (expression atlas of Arabidopsis development); (B) Different stages of seed development (original repository: ArrayExpress (E-GEOD-5634); (C) Somatic embryos after 10 d of culture (original repository: GEO (GSE17610)). [file 12864_2015_1504_MOESM5_ESM.pdf]

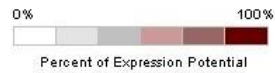

**Arabidopsis thaliana (45)**

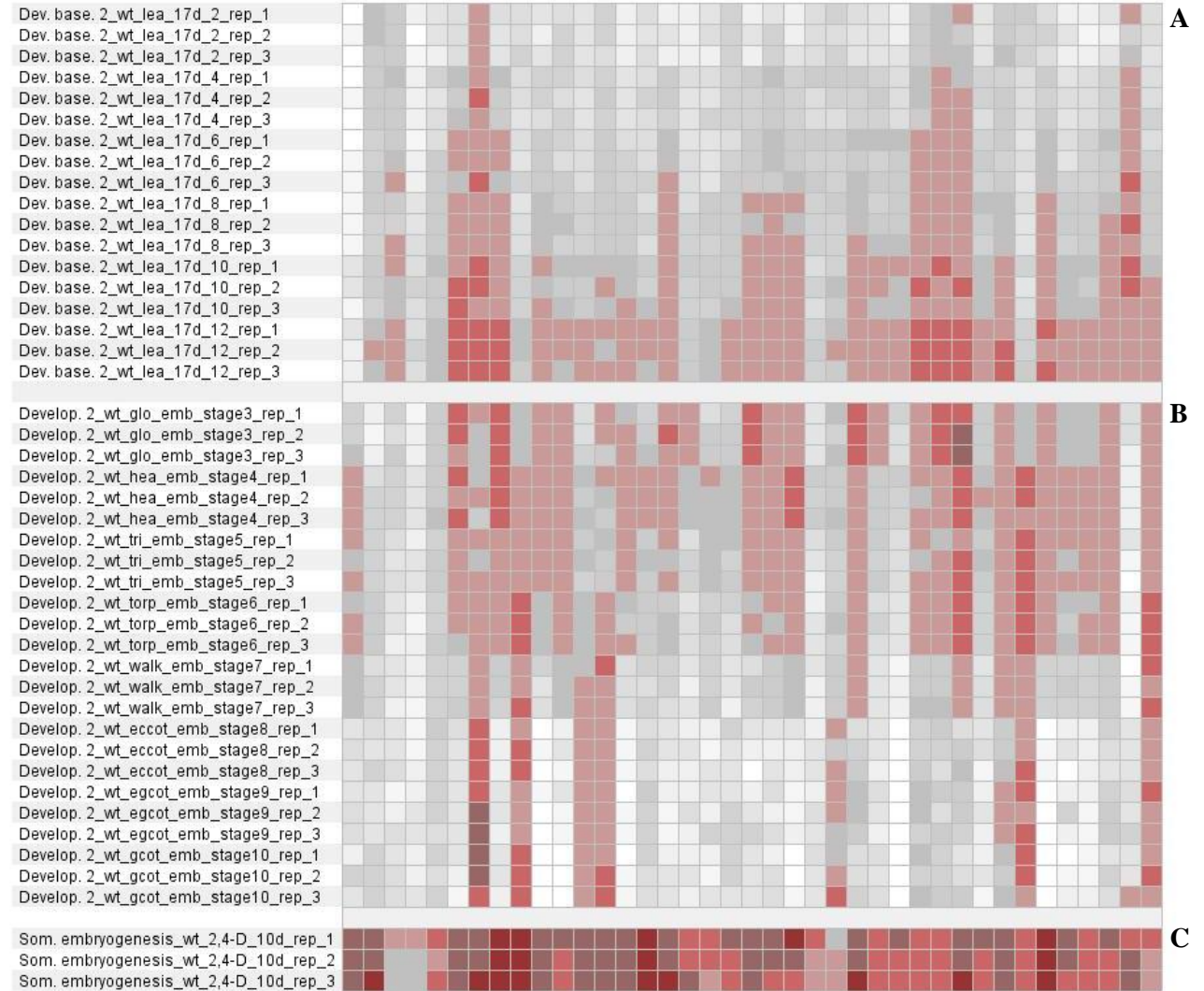

Supplement: Additional file 6: — Expression data extracted from the Genevestigator for a subset of genes related to DNA replication. (A) Mature leaf tissue (original repository: AtGenExpress (expression atlas of Arabidopsis development); (B) Different stages of seed development (original repository: ArrayExpress (E-GEOD-5634); (C) Somatic embryos after 10 d of culture (original repository: GEO (GSE17610)). [file 12864_2015_1504_MOESM6_ESM.pdf]

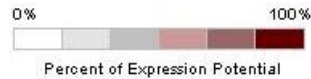

**Arabidopsis thaliana (45)**

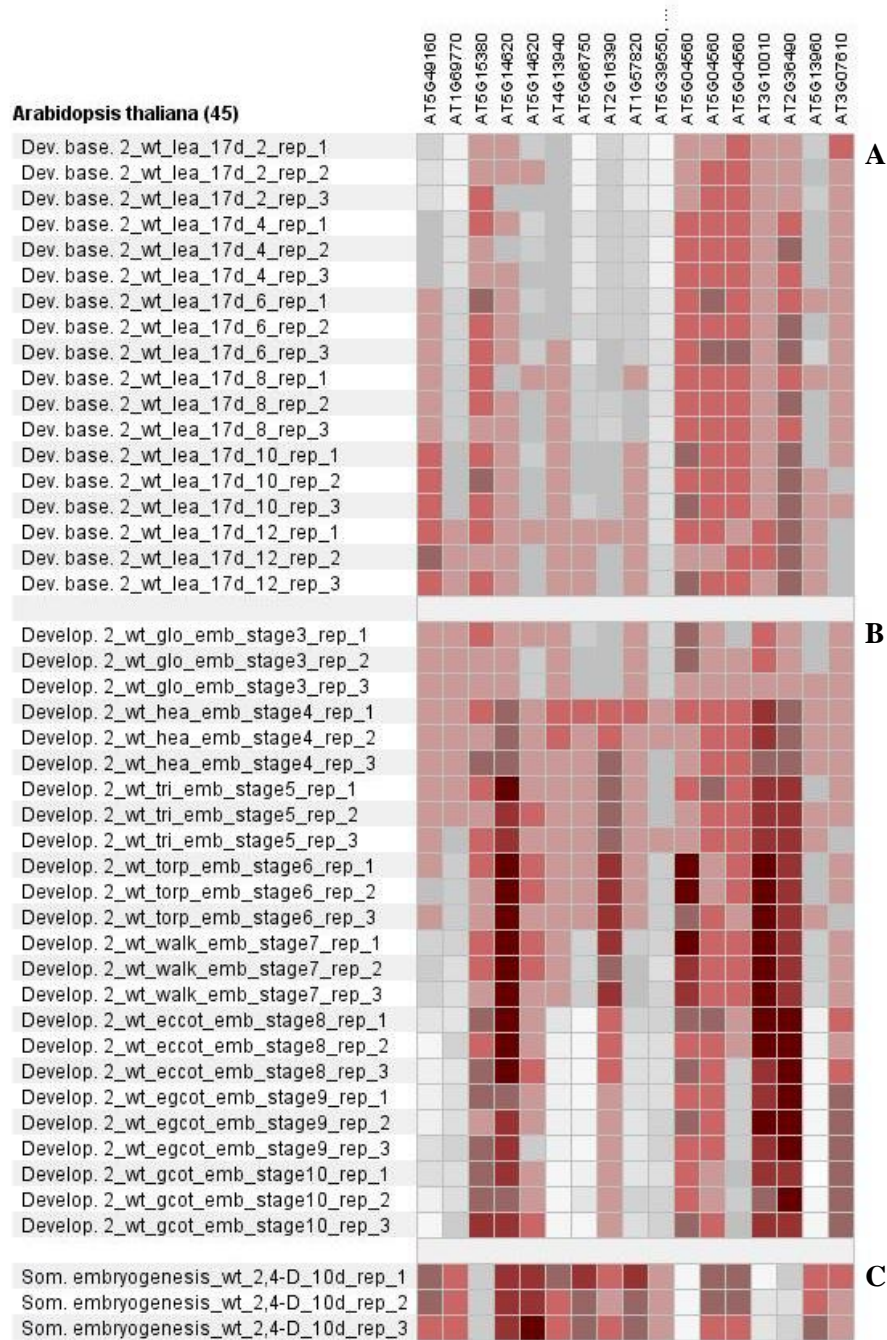

Supplement: Additional file 7: — Expression data extracted from the Genevestigator for a subset of genes related to DNA cytosine methylation. (A) Mature leaf tissue (original repository: AtGenExpress (expression atlas of Arabidopsis development); (B) Different stages of seed development (original repository: ArrayExpress (E-GEOD-5634); (C) Somatic embryos after 10 d of culture (original repository: GEO (GSE17610)). [file 12864_2015_1504_MOESM7_ESM.pdf]

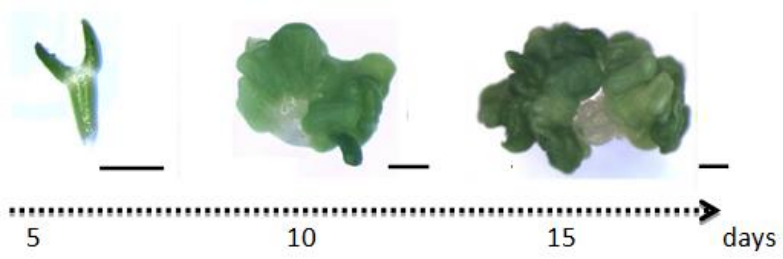

Supplement: Additional file 11: — Time-points selected for tissue sampling and total RNA extraction from IZE explants cultured on B5 medium supplemented with 5 μM 2, 4-D. Scale bars = 200 μm. [file 12864_2015_1504_MOESM11_ESM.pdf]
