# Supplementary material for: Global scale transcriptome analysis of Arabidopsis embryogenesis in vitro
Source: BMC Genomics. 2015 Apr 16;16(1):301. doi: 10.1186/s12864-015-1504-6 (PMC4404573; doi:10.1186/s12864-015-1504-6)
Supplement: Additional file 3: — List of potential candidate genes for molecular studies. This subset of genes was prepared from a list of more highly expressed genes detected in somatic embryos in the present study which had links to known mutant seed phenotypes (SeedGenes database). [file 12864_2015_1504_MOESM3_ESM.pdf]

Chromosome locus	Gene symbol	Allele symbol	Gene class	Terminal phenotype	Location of mutation
AT1G01370	*CENH 3*	cenh 3-1	Embryo defective	Not available	
AT1G02780	*EMB 2386*	emb 2386	Embryo defective	Globular	Intron 1
AT1G03360	*RRP 4*	rrp 4-1	Embryo defective	Preglobular	Promoter
AT1G04635	*EMB 1687*	emb 1687-1	Embryo defective	Globular	Exon 1
AT1G04635	*EMB 1687*	emb 1687-2	Embryo defective	Globular	Exon 1
AT1G08130	*LIG 1*	lig 1	Embryo defective	Not available	Intron
AT1G08260	*EMB 2284*	emb 2284-1	Embryo defective	Globular	Intron 44
AT1G08260	*EMB 2284*	emb 2284-2	Embryo defective	Globular	Intron 47 to Exon 48
AT1G08260	*EMB 2284*	emb 2284-3	Embryo defective	Globular	Intron 7
AT1G08260	*EMB 2284*	emb 529	Embryo defective	Globular	Intron 25
AT1G08260	*EMB 2284*	emb 2284-5	Embryo defective	Globular	5' UTR
AT1G08260	*EMB 2284*	til 1-4	Embryo defective	Cotyledon	Exon 12
AT1G08260	*EMB 2284*	emb 142	Embryo defective	Globular	Exon 3
AT1G08560	*KN*	kn	Embryo defective	Cotyledon	
AT1G10270	*GRP 23*	grp 23-1	Embryo defective	Preglobular	Exon 1
AT1G10270	*GRP 23*	grp 23-2	Embryo defective	Not available	Exon 1
AT1G10270	*GRP 23*	grp 23-3	Embryo defective	Not available	Exon 1
AT1G18450	*ARP 4*	arp 4-3	Embryo defective	Not available	Intron 1
AT1G19850	*MP*	mp	Embryo defective	Cotyledon	
AT1G21310	*RSH*	rsh	Embryo defective	Cotyledon	Upstream
AT1G21690	*EMB 1968*	emb 1968-1	Embryo defective	Preglobular	Intron 3
AT1G21690	*EMB 1968*	emb 1968-2	Embryo defective	Preglobular	Intron 2
AT1G21690	*EMB 1968*	emb 1968-3	Embryo defective	Preglobular	Exon 2
AT1G21970	*LEC 1*	lec 1-1	Embryo defective	Cotyledon	Entire gene deleted
AT1G21970	*LEC 1*	lec 1-2	Embryo defective	Cotyledon	Upstream
AT1G28300	*LEC 2*	lec 2-1	Embryo defective	Cotyledon	Deletion 5' to exon 2
AT1G31860	*HISN 2*	hisn 2-1	Embryo defective	Preglobular	Exon 5
AT1G31860	*HISN 2*	hisn 2-2	Embryo defective	Preglobular	Exon 5
AT1G31860	*HISN 2*	hisn 2-3	Embryo defective	Cotyledon	5' UTR
AT1G43170	*EMB 2207*	emb 2207-1	Embryo defective	Globular	Exon 5
AT1G43170	*EMB 2207*	emb 2207-2	Embryo defective	Globular	Exon 5
AT1G44900	*MCM 2*	mcm 2-1	Embryo defective	Preglobular	Exon 7
AT1G44900	*MCM 2*	mcm 2-2	Embryo defective	Preglobular	Intron 1
AT1G49400	*EMB 1129*	emb 1129	Embryo defective	Preglobular	Upstream to 5' UTR
AT1G55600	*MINI 3*	mini 3-1	Embryo defective	Cotyledon	Exon 5
AT1G55600	*MINI 3*	mini 3-2	Embryo defective	Cotyledon	Exon 3
AT1G55900	*EMB 1860*	emb 1860-1	Embryo defective	Preglobular	Intron 9 to 3' UTR

AT1G55900	*EMB 1860*	emb 1860-2	Embryo defective	Not available	Exon 5
AT1G62360	*STM*	stm-1	Embryo defective	Not available	
AT1G62360	*STM*	stm-3	Embryo defective	Not available	
AT1G63160	*EMB 2811*	emb 2811-1	Embryo defective	Preglobular	Exon 5
AT1G63160	*EMB 2811*	emb 2811-2	Embryo defective	Preglobular	Exon 4
AT1G67320	*EMB 2813*	emb 2813-1	Embryo defective	Preglobular	Exon 8
AT1G67320	*EMB 2813*	emb 2813-2	Embryo defective	Preglobular	Intron 12
AT1G67630	*EMB 2814*	emb 2814-1	Embryo defective	Preglobular	Intron 7
AT1G67630	*EMB 2814*	emb 2814-2	Embryo defective	Preglobular	Exon 4
AT1G68450	*PDE 337*	pde 337	Seed pigment	Pigment defective	Exon 1
AT1G77470	*EMB 2810*	emb 2810	Embryo defective	Preglobular	Intron 3
AT1G79490	*EMB 2217*	emb 2217	Embryo defective	Globular	Exon 1
AT2G01140	*PDE 345*	pde 345-1	Seed pigment	Pigment defective	Exon 2
AT2G01140	*PDE 345*	pde 345-2	Seed pigment	Pigment defective	Promoter or exon 4
AT2G17090	*SSP*	ssp	Embryo defective	Preglobular	Kinase domain
AT2G17250	*EMB 2762*	emb 2762-1	Embryo defective	Globular	Intron 7
AT2G17250	*EMB 2762*	emb 2762-2	Embryo defective	Globular	Intron 13
AT2G18020	*EMB 2296*	emb 2296-1	Embryo defective	Globular	Exon 2
AT2G18020	*EMB 2296*	emb 2296-2	Embryo defective	Globular	Exon 2 to 3' UTR
AT2G18390	*TTN 5*	ttn 5-1	Embryo defective	Preglobular	Intron 3 to exon 4
AT2G18390	*TTN 5*	ttn 5-2	Embryo defective	Preglobular	Exon 3
AT2G19450	*TAG 1*	tag 1-1	Embryo defective	Cotyledon	Intron 2
AT2G19450	*TAG 1*	tag 1-2	Embryo defective	Cotyledon	Exon 1
AT2G31060	*EMB 2785*	emb 2785-1	Embryo defective	Globular	Exon 9 or 11
AT2G31060	*EMB 2785*	emb 2785-2	Embryo defective	Globular	Intron 15 or 17
AT2G32590	*EMB 2795*	emb 2795-1	Embryo defective	Preglobular	Intron 9
AT2G32590	*EMB 2795*	emb 2795-2	Embryo defective	Preglobular	Intron 12 or exon 13
AT2G34650	*PID*	pid-3	Embryo defective	Cotyledon	Exon 2
AT2G34650	*PID*	pid-1	Embryo defective	Cotyledon	Exon 1
AT2G35670	*FIS 2*	fis 2	50% Defective seeds	Cotyledon	
AT2G37560	*ORC 2*	orc 2	Embryo defective	Globular	Exon 6
AT2G38770	*EMB 2765*	emb 2765-1	Embryo defective	Globular	Exon 1
AT2G38770	*EMB 2765*	emb 2765-2	Embryo defective	Globular	Intron 6
AT2G43650	*EMB 2777*	emb 2777	Embryo defective	Cotyledon	Exon 13
AT2G44190	*EMB 3116*	emb 3116-1	Embryo defective	Preglobular	Exon 1
AT2G44190	*EMB 3116*	emb 3116-2	Embryo defective	Preglobular	Exon 1
AT2G44190	*EMB 3116*	ede 1-1	Embryo defective	Cotyledon	Exon 3

AT2G44190	*EMB 3116*	ede 1-2	Embryo defective	Transition	Exon 1
AT2G44190	*EMB 3116*	ede 1-3	Embryo defective	Transition	Intron 3
AT2G45000	*EMB 2766*	emb 2766-1	Embryo defective	Cotyledon	Intron 5
AT2G45000	*EMB 2766*	emb 2766-2	Embryo defective	Cotyledon	Intron 5
AT3G04400	*EMB 2171*	emb 2171	Embryo defective	Globular	5' UTR
AT3G07060	*EMB 1974*	emb 1974-1	Embryo defective	Cotyledon	Intron 9
AT3G07060	*EMB 1974*	emb 1974-2	Embryo defective	Cotyledon	Intron 4
AT3G11940	*AML 1*	aml 1	Embryo defective	Globular	Exon 5
AT3G12670	*EMB 2742*	emb 2742-1	Embryo defective	Globular	Intron 5
AT3G12670	*EMB 2742*	emb 2742-2	Embryo defective	Globular	Upstream
AT3G17910	*EMB 3121*	emb 3121-1	Embryo defective	Cotyledon	
AT3G17910	*EMB 3121*	emb 3121-2	Embryo defective	Cotyledon	Intron 1
AT3G19700	*IKU 2*	iku 2-3	Embryo defective	Cotyledon	Kinase domain
AT3G19700	*IKU 2*	iku 2-1	Embryo defective	Cotyledon	Amino acid 49
AT3G19700	*IKU 2*	iku 2-2	Embryo defective	Cotyledon	Deletion / frameshift
AT3G20400	*EMB 2743*	emb 2743	Embryo defective	Cotyledon	Downstream
AT3G20440	*EMB 2729*	emb 2729-1	Embryo defective	Cotyledon	Intron 21
AT3G20440	*EMB 2729*	emb 2729-2	Embryo defective	Transition	Intron 15
AT3G20630	*TTN 6*	ttn 6-1	Embryo defective	Globular	Intron 10 to downstream
AT3G20630	*TTN 6*	ttn 6-4	Embryo defective	Globular	Intron 5 to intron 7
AT3G26790	*FUS 3*	fus 3-1	Embryo defective	Cotyledon	Intron 3
AT3G46560	*EMB 2474*	emb 2474	Embryo defective	Preglobular	Exon 2
AT3G48930	*EMB 1080*	emb 1080	Embryo defective	Globular	Exon 4
AT3G49240	*EMB 1796*	emb 1796-1	Embryo defective	Globular	3' UTR to downstream
AT3G49240	*EMB 1796*	emb 1796-2	Embryo defective	Globular	Exon 1
AT3G49660	*AtMUT 11*	atmut 11	Embryo defective	Not available	
AT3G50870	*MNP*	mnp	Embryo defective	Cotyledon	
AT3G52590	*EMB 2167*	emb 2167-1	Embryo defective	Transition	5' UTR
AT3G52590	*EMB 2167*	emb 2167-3	Embryo defective	Globular	Intron 4 to exon 5
AT3G52590	*EMB 2167*	emb 2167-4	Embryo defective	Globular	Intron 3
AT3G54320	*WRI 1*	wri 1-1	Embryo defective	Cotyledon	Intron 1
AT3G54650	*FBL 17*	fbl 17-1	50% Defective seeds	Globular	Exon 7
AT3G54650	*FBL 17*	fbl 17-2	50% Defective seeds	Globular	Intron 6
AT3G55010	*EMB 2818*	emb 2818-1	Embryo defective	Preglobular	Intron 3
AT3G55010	*EMB 2818*	emb 2818-2	Embryo defective	Preglobular	Intron 7
AT3G55510	*RBL*	rbl-3	Embryo defective	Transition	Exon 6
AT4G00620	*EMB 3127*	emb 3127-1	Embryo defective	Preglobular	Exon 1
AT4G00620	*EMB 3127*	emb 3127-2	Embryo defective	Preglobular	Exon 1
AT4G02060	*PRL*	prl	Embryo defective	Globular	Intron 3

AT4G05410	*YAO*	yao	Embryo defective	Preglobular	Exon 4
AT4G13750	*EMB 2597*	emb 2597-1	Embryo defective	Cotyledon	Upstream to exon 1
AT4G13750	*EMB 2597*	emb 2597-2	Embryo defective	Cotyledon	Exon 1
AT4G13940	*EMB 1395*	emb 1395	Embryo defective	Globular	Intron 1
AT4G13940	*EMB 1395*	hog 1-4	Embryo defective	Globular	Exon 1
AT4G13940	*EMB 1395*	hog 1-5	Embryo defective	Globular	Exon 2
AT4G14110	*COP 9*	cop 9-1	Seed pigment	Fusca	Exon 3
AT4G22970	*AESP*	aesp-1	Embryo defective	Globular	Exon 1
AT4G22970	*AESP*	aesp-2	Embryo defective	Globular	Intron 17
AT4G24270	*EMB 140*	emb 140	Embryo defective	Globular	Exon 2
AT4G27010	*EMB 2788*	emb 2788-1	Embryo defective	Cotyledon	Exon 1
AT4G27010	*EMB 2788*	emb 2788-2	Embryo defective	Cotyledon	Exon 2
AT4G32400	*EMB 104*	emb 104-1	Embryo defective	Cotyledon	Exon 3
AT4G32400	*EMB 104*	emb 104-2	Embryo defective	Cotyledon	
AT4G32400	*EMB 104*	emb 104-3	Embryo defective	Transition	Intron 2
AT4G32400	*EMB 104*	emb 104-4	Embryo defective	Globular	Exon 3
AT4G32720	*AtLA 1*	atla 1-1	Embryo defective	Globular	Exon 8
AT4G32720	*AtLA 1*	atla 1-2	Embryo defective	Globular	Exon 8
AT4G33990	*EMB 2758*	emb 2758	Embryo defective	Globular	Exon 1
AT5G05560	*EMB 2771*	emb 2771	Embryo defective	Cotyledon	Exon
AT5G05680	*EMB 2789*	emb 2789-1	Embryo defective	Preglobular	Exon 6
AT5G05680	*EMB 2789*	emb 2789-2	Embryo defective	Globular	Intron 3
AT5G07500	*PEI*	pei	Embryo defective	Cotyledon	
AT5G08610	*PDE 340*	pde 340	Seed pigment	Pigment Defective	Exon 1
AT5G09790	*PDE 336*	pde 336	Seed pigment	Pigment Defective	Exon 1
AT5G10360	*EMB 3010*	emb 3010	Embryo defective	Preglobular	Exon 5
AT5G14800	*EMB 2772*	emb 2772-1	Embryo defective	Preglobular	Exon 1
AT5G14800	*EMB 2772*	emb 2772-2	Embryo defective	Preglobular	Intron 5
AT5G15920	*EMB 2782*	emb 2782-1	Embryo defective	Preglobular	Exon 1
AT5G15920	*EMB 2782*	emb 2782-2	Embryo defective	Preglobular	Exon 1
AT5G16750	*TOZ*	toz	Embryo defective	Preglobular	
AT5G18700	*EMB 3013*	emb 3013	Embryo defective	Cotyledon	Intron 9
AT5G18820	*EMB 3007*	emb 3007	Embryo defective	Globular	Intron 7
AT5G20920	*EMB 1401*	emb 1401	Embryo defective	Cotyledon	Upstream to exon 1 in 5' UTR
AT5G21140	*EMB 1379*	emb 1379-1	Embryo defective	Preglobular	Intron 1
AT5G21140	*EMB 1379*	emb 1379-2	Embryo defective	Globular	Intron 3
AT5G22110	*CYL 2*	cyl 2	Embryo defective	Globular	Exon 1
AT5G23880	*EMB 1265*	emb 1265	Embryo defective	Preglobular	Intron 8
AT5G23940	*EMB 3009*	emb 3009-1	Embryo defective	Preglobular	Intron 1
AT5G23940	*EMB 3009*	emb 3009-2	Embryo defective	Not available	
AT5G37630	*EMB 2656*	emb 2656-1	Embryo defective	Preglobular	Intron 7

AT5G37630	*EMB 2656*	emb 2656-2	Embryo defective	Preglobular	Intron 3
AT5G40480	*EMB 3012*	emb 3012	Embryo defective	Preglobular	Intron 17
AT5G48600	*SMC 4*	smc 4	Embryo defective	Globular	Intron 7
AT5G48720	*XRI 1*	xri 1-1	50% Defective seeds	Preglobular	Exon 3
AT5G49010	*EMB 2812*	emb 2812	Embryo defective	Not available	Intron 2
AT5G49160	*MET 1*	met 1-6	Embryo defective	Cotyledon	
AT5G51200	*EMB 3142*	emb 3142	Embryo defective	Preglobular	
AT5G53400	*BOB 1*	bob 1-1	Embryo defective	Globular	Exon 3
AT5G53400	*BOB 1*	bob 1-2	Embryo defective	Globular	Exon 1
AT5G59440	*ZEUS 1*	zeus 1	Embryo defective	Preglobular	Gene deletion
AT5G62410	*TTN 3*	ttn 3	Embryo defective	Cotyledon	Intron 14
AT5G62440	*DOM 1*	dom 1-1	Embryo defective	Globular	Intron 1
AT5G62440	*DOM 1*	dom 1-2	Embryo defective	Globular	Intron 1
