# Supplementary material for: Global scale transcriptome analysis of Arabidopsis embryogenesis in vitro
Source: BMC Genomics. 2015 Apr 16;16(1):301. doi: 10.1186/s12864-015-1504-6 (PMC4404573; doi:10.1186/s12864-015-1504-6)
Supplement: Additional file 4: — Expression data extracted from the Genevestigator for a subset of highly expressed genes detected in WT leaf than in somatic embryos. (A) Mature leaf tissue (original repository: AtGenExpress (expression atlas of Arabidopsis development); (B) Different stages of seed development (original repository: ArrayExpress (E-GEOD-5634); (C) Somatic embryos after 10 d of culture (original repository: GEO (GSE17610)). [file 12864_2015_1504_MOESM4_ESM.pdf]

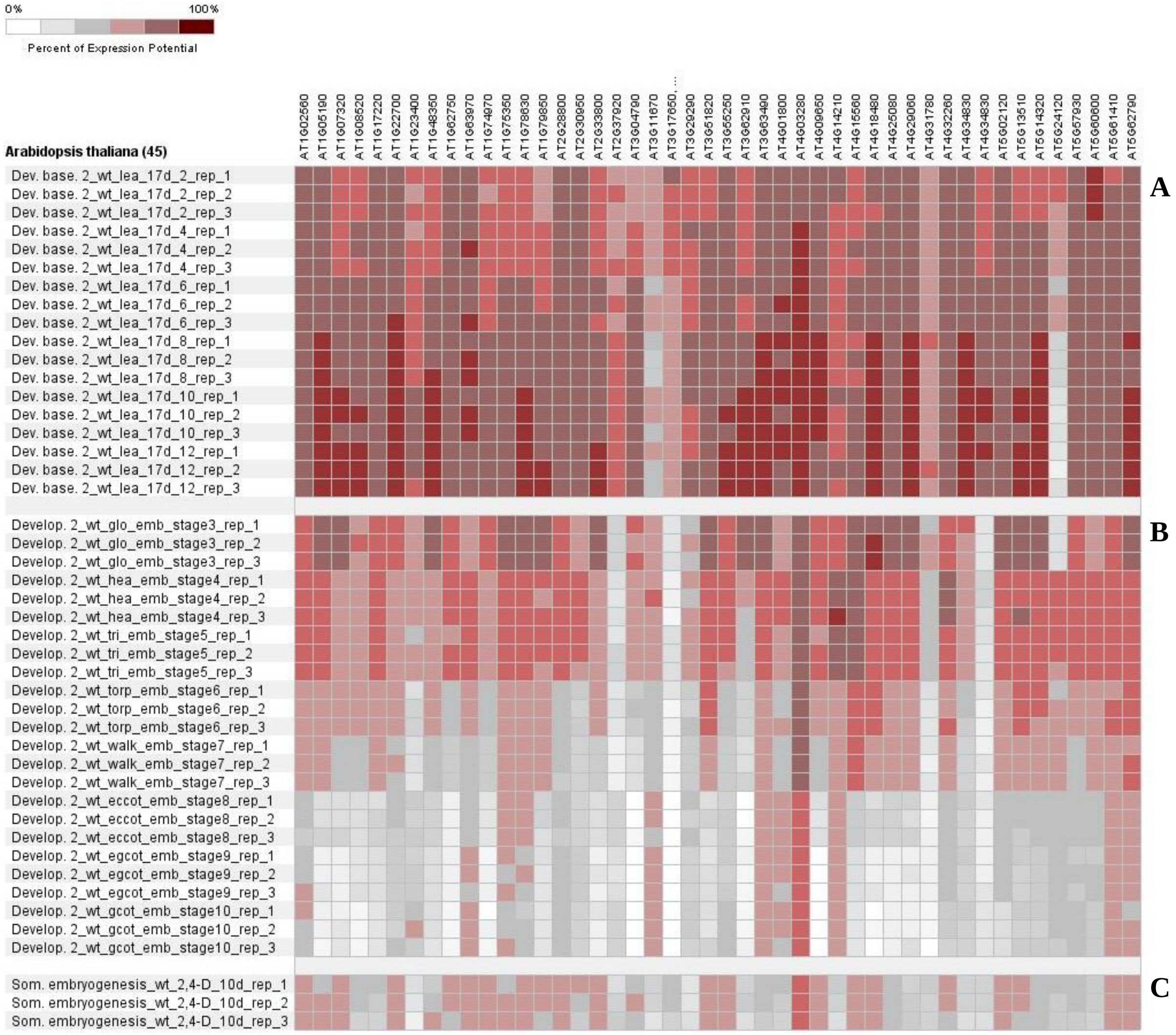
0%
100%
Percent of Expression Potential
Arabidopsis thaliana (45)
AT1G02560
AT1G05190
AT1G07320
AT1G08520
AT1G17220
AT1G22700
AT1G23400
AT1G48350
AT1G62750
AT1G63970
AT1G74970
AT1G75350
AT1G78630
AT1G79850
AT2G28800
AT2G30950
AT2G33800
AT2G37920
AT3G04790
AT3G11670
AT3G17650, ...
AT3G29290
AT3G51820
AT3G55250
AT3G62910
AT3G63490
AT4G01800
AT4G03280
AT4G09650
AT4G14210
AT4G15560
AT4G18480
AT4G25080
AT4G29060
AT4G31780
AT4G32260
AT4G34830
AT4G34830
AT5G02120
AT5G13510
AT5G14320
AT5G24120
AT5G57930
AT5G60600
AT5G61410
AT5G62790
Dev. base. 2_wt_lea_17d_2_rep_1
Dev. base. 2_wt_lea_17d_2_rep_2
Dev. base. 2_wt_lea_17d_2_rep_3
Dev. base. 2_wt_lea_17d_4_rep_1
Dev. base. 2_wt_lea_17d_4_rep_2
Dev. base. 2_wt_lea_17d_4_rep_3
Dev. base. 2_wt_lea_17d_6_rep_1
Dev. base. 2_wt_lea_17d_6_rep_2
Dev. base. 2_wt_lea_17d_6_rep_3
Dev. base. 2_wt_lea_17d_8_rep_1
Dev. base. 2_wt_lea_17d_8_rep_2
Dev. base. 2_wt_lea_17d_8_rep_3
Dev. base. 2_wt_lea_17d_10_rep_1
Dev. base. 2_wt_lea_17d_10_rep_2
Dev. base. 2_wt_lea_17d_10_rep_3
Dev. base. 2_wt_lea_17d_12_rep_1
Dev. base. 2_wt_lea_17d_12_rep_2
Dev. base. 2_wt_lea_17d_12_rep_3
A
Develop. 2_wt_glo_emb_stage3_rep_1
Develop. 2_wt_glo_emb_stage3_rep_2
Develop. 2_wt_glo_emb_stage3_rep_3
Develop. 2_wt_hea_emb_stage4_rep_1
Develop. 2_wt_hea_emb_stage4_rep_2
Develop. 2_wt_hea_emb_stage4_rep_3
Develop. 2_wt_tri_emb_stage5_rep_1
Develop. 2_wt_tri_emb_stage5_rep_2
Develop. 2_wt_tri_emb_stage5_rep_3
Develop. 2_wt_torp_emb_stage6_rep_1
Develop. 2_wt_torp_emb_stage6_rep_2
Develop. 2_wt_torp_emb_stage6_rep_3
Develop. 2_wt_walk_emb_stage7_rep_1
Develop. 2_wt_walk_emb_stage7_rep_2
Develop. 2_wt_walk_emb_stage7_rep_3
Develop. 2_wt_eccot_emb_stage8_rep_1
Develop. 2_wt_eccot_emb_stage8_rep_2
Develop. 2_wt_eccot_emb_stage8_rep_3
Develop. 2_wt_egcot_emb_stage9_rep_1
Develop. 2_wt_egcot_emb_stage9_rep_2
Develop. 2_wt_egcot_emb_stage9_rep_3
Develop. 2_wt_gcot_emb_stage10_rep_1
Develop. 2_wt_gcot_emb_stage10_rep_2
Develop. 2_wt_gcot_emb_stage10_rep_3
B
Som. embryogenesis_wt_2,4-D_10d_rep_1
Som. embryogenesis_wt_2,4-D_10d_rep_2
Som. embryogenesis_wt_2,4-D_10d_rep_3
C
