# Supplementary material for: Global scale transcriptome analysis of Arabidopsis embryogenesis in vitro
Source: BMC Genomics. 2015 Apr 16;16(1):301. doi: 10.1186/s12864-015-1504-6 (PMC4404573; doi:10.1186/s12864-015-1504-6)
Supplement: Additional file 8: — Hierarchical clustering (using Euclidian distance measure) for a subset of 100 randomly selected genes, using microarray expression data from somatic embryos (AT-00508), callus (AT-00265) and leaf (AT-00265) in Arabidopsis. The gene list employed in this study was selected from the list of more highly expressed genes identified in somatic embryos in the present study. Genevestigator hierarchical clustering tool was used to construct the clustering tree. The genes more highly expressed in somatic embryos as compared to leaf and callus tissues are highlighted in yellow (Log2 [FC] ≥ 2.0). Orange, blue and purple colours highlight the samples from somatic embryos, callus and leaf tissues, respectively. [file 12864_2015_1504_MOESM8_ESM.pdf]

Dataset: 6 samples (sample selection: AT-SAMPLES-0)

100 genes (gene selection: AT-GENES-1)

0% 100%

Percent of Expression Potential

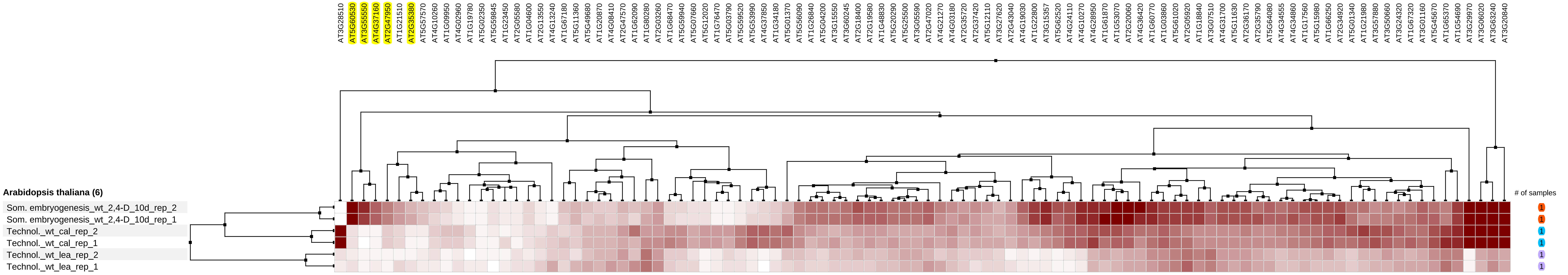

created with GENEVESTIGATOR
