# Supplementary material for: Global scale transcriptome analysis of Arabidopsis embryogenesis in vitro
Source: BMC Genomics. 2015 Apr 16;16(1):301. doi: 10.1186/s12864-015-1504-6 (PMC4404573; doi:10.1186/s12864-015-1504-6)
Supplement: Additional file 9: — Description of the 49 genes that exhibited a higher level of expression in somatic embryos (AT-00508) than in callus (AT-00265), torpedo stage zygotic embryos (AT-00629) or leaf tissues (AT-00265), based on microarray data extracted from the Genevestigator tool. [file 12864_2015_1504_MOESM9_ESM.pdf]

Gene ID	Gene description
AT1G02900	Member of a diversely expressed predicted peptide family showing sequence similarity to tobacco Rapid Alkalinization Factor (RALF), and is believed to play an essential role in the physiology of Arabidopsis
AT1G03790	Encodes SOMNUS (SOM), a nucleus-localized CCCH-type zinc finger protein. SOM negatively regulates light-dependent seed germination downstream of PIL5 (AT2G20180); Zinc finger C-x8-C-x5-C-x3-H type family protein
AT1G14080	Member of Xyloglucan fucosyltransferase family; fucosyltransferase 6
AT1G47400	Unknown protein
AT1G50060	CAP (Cysteine-rich secretory proteins, Antigen 5, and Pathogenesis-related 1 protein) superfamily protein
AT1G52910	Protein of unknown function (DUF1218)
AT1G59730	Thioredoxin H-type 7 (TH7)
AT1G64405	Unknown protein
AT1G68850	Peroxidase superfamily protein
AT1G72230	Cupredoxin superfamily protein
AT1G77330	Similar to 1-aminocyclopropane-1-carboxylate oxidase GI:3386565 from (Sorghum bicolor);2-oxoglutarate (2OG) and Fe(II)-dependent oxygenase superfamily protein
AT2G03200	Eukaryotic aspartyl protease family protein
AT2G14095	Unknown protein
AT2G18370	Predicted to encode a PR (pathogenesis-related) protein; bifunctional inhibitor/lipid-transfer protein/seed storage 2S albumin superfamily protein
AT2G18980	Peroxidase superfamily protein
AT2G25810	tonoplast intrinsic protein 4;1 (TIP4;1)
AT2G28870	Unknown protein
AT2G41850	ADPG2; polygalacturonase abscission zone *A. thaliana*
AT2G43590	Chitinase family protein
AT2G47200	Unknown protein

AT3G16440	myrosinase-binding protein-like protein (AtMLP-300B) mRNA; myrosinase-binding protein-like protein-300B
AT3G17380	TRAF-like family protein
AT3G19430	Late embryogenesis abundant protein-related / LEA protein-related
AT3G22620	Bifunctional inhibitor/lipid-transfer protein/seed storage 2S albumin superfamily protein
AT3G46280	Protein kinase-related
AT4G01240	S-adenosyl-L-methionine-dependent methyltransferases superfamily protein
AT4G02330	Plant invertase/pectin methylesterase inhibitor superfamily
AT4G11610	C2 calcium/lipid-binding plant phosphoribosyltransferase family protein
AT4G12470	Encodes AZI1 (AZELAIC ACID INDUCED 1)
AT4G27400	Late embryogenesis abundant (LEA) protein-related
AT4G28720	Auxin biosynthetic gene; Flavin-binding monooxygenase family protein
AT4G30140	GDSL-like Lipase/Acylhydrolase superfamily protein
AT4G37160	SKU5 similar 15 (sks15)
AT5G02640	Unknown protein
AT5G13880	Unknown protein
AT5G14130	Peroxidase superfamily protein
AT5G17820	Peroxidase superfamily protein
AT5G22460	alpha/beta-Hydrolases superfamily protein
AT5G40510	Sucrase/ferredoxin-like family protein
AT5G44380	FAD-binding Berberine family protein
AT5G51470	Auxin-responsive GH3 family protein

AT5G52390	PAR1 protein
AT5G54370	Late embryogenesis abundant (LEA) protein-related
AT5G58860	Encodes a member of the CYP86A subfamily of cytochrome p450 genes
AT5G60530	Late embryogenesis abundant protein-related / LEA protein-related
AT5G61650	The P-type cyclins (CYCPs); CYCLIN P4;2
AT5G62340	Plant invertase/pectin methylesterase inhibitor superfamily protein
AT5G64120	Encodes a cell wall bound peroxidase that is induced by hypo-osmolarity; Peroxidase superfamily protein
AT5G65320	basic helix-loop-helix (bHLH) DNA-binding superfamily protein
